# Dynamic Reconfiguration of Hippocampal Interneuron Circuits during Spatial Learning

**DOI:** 10.1016/j.neuron.2013.01.033

**Published:** 2013-04-10

**Authors:** David Dupret, Joseph O’Neill, Jozsef Csicsvari

**Affiliations:** 1 MRC Anatomical Neuropharmacology Unit, Department of Pharmacology, University of Oxford, Mansfield Road, Oxford OX1 3TH, UK; 2 IST Austria, Am Campus 1, A–3400 Klosterneuburg, Austria

## Abstract

In the hippocampus, cell assemblies forming mnemonic representations of space are thought to arise as a result of changes in functional connections of pyramidal cells. We have found that CA1 interneuron circuits are also reconfigured during goal-oriented spatial learning through modification of inputs from pyramidal cells. As learning progressed, new pyramidal assemblies expressed in theta cycles alternated with previously established ones, and eventually overtook them. The firing patterns of interneurons developed a relationship to new, learning-related assemblies: some interneurons associated their activity with new pyramidal assemblies while some others dissociated from them. These firing associations were explained by changes in the weight of monosynaptic inputs received by interneurons from new pyramidal assemblies, as these predicted the associational changes. Spatial learning thus engages circuit modifications in the hippocampus that incorporate a redistribution of inhibitory activity that might assist in the segregation of competing pyramidal cell assembly patterns in space and time.

## Highlights

► Interneuron firing reorganizes as pyramidal assemblies flicker during spatial learning ► Interneurons form dynamic firing associations to newly established assemblies ► Changes in pyramidal cell connections to interneurons explain firing associations ► Pyramidal cell-interneuron coupling changes are activity dependent and map specific

## Teaser

Dupret et al. show that CA1 hippocampal interneuron circuits undergo plastic changes during spatial learning, enabling them to develop firing associations with newly established pyramidal cell assemblies. These learning-related circuit modifications may assist in the segregation of competing pyramidal assemblies.

## Introduction

Current theories of memory formation suggest that experience-dependent modifications of synaptic weights enable a selected group of neurons to form new associations, leading to the establishment of new cell assemblies to represent mnemonic information (Buzsáki, 2010; Martin and Morris, 2002). In the hippocampus, principal cells encode the current location of the animal, allowing different cell assemblies to represent different locations (Leutgeb et al., 2005; O’Keefe and Dostrovsky, 1971; Wilson and McNaughton, 1993). Such hippocampal representations develop when the animal is placed into a new environment, so that each new environment explored is represented by different sets of cell assemblies that comprise a unique “cognitive map” of the allocentric space (Moser et al., 2008; Muller, 1996; O’Keefe and Nadel, 1978). In addition to forming new maps of previously unseen environments, this “remapping” also occurs in conjunction with spatial learning, even in a familiar environment, raising the possibility that the formation of spatial memory traces involve the reorganization of cell assembly patterns. Indeed, in the CA1 region, new place maps are established during reward-associated spatial learning, resulting in the formation of new cell assemblies that represent information about the locations of food resources (Dupret et al., 2010).

The detailed temporal dynamics that facilitate the development of new maps during spatial learning remain to be examined. Although it is expected that new maps undergo a process of refinement, it is not clear whether the old maps associated with previous learning episodes are temporarily retained during the learning. Recently it has been discovered that cell assembly patterns can flicker rapidly between the representation of different maps across consecutive theta oscillatory cycles when environmental cues or task parameters are abruptly changed (Jackson and Redish, 2007; Jezek et al., 2011; Kelemen and Fenton, 2010). It is possible that such flickering may also take place between old and newly-formed representations during spatial learning. This could enable competitive processes in which old and new maps initially vie for prominence, with the new maps dominating in later stages of learning. Such competitive network dynamics may be an integral part of spatial learning and map refinement, allowing for effective behavioral adaptation in response to the environment.

Inhibitory interneurons may prove to be instrumental in spatial learning and dynamic behaviorally adaptive network process. Indeed, it has recently been suggested that interneurons might assist in the organization of pyramidal cell assemblies during learning (Assisi et al., 2011; Buzsáki, 2010). For instance, the abrupt change of interneuron firing rates observed while the animal is exposed to a novel environment could promote the formation of new maps and the associated reorganization of pyramidal assemblies (Frank et al., 2004; Nitz and McNaughton, 2004; Wilson and McNaughton, 1993). If interneurons have a role in shaping pyramidal cell assemblies, it is possible that spatial learning and the associated formation of new pyramidal assemblies may be accompanied by alterations in interneuron circuitry as well. One possible circuit change may occur on local pyramidal inputs targeting interneurons, which itself could contribute to the interneuron firing rate changes during spatial learning. Indeed, glutamatergic synapses targeting GABAergic interneurons in the hippocampus are modifiable in an activity-dependent manner (Alle et al., 2001; Lamsa et al., 2005, 2007; Perez et al., 2001). Given that a single presynaptic pyramidal cell can reliably excite its postsynaptic interneurons in the hippocampus, the modification of pyramidal cell-interneuron connections can exert wide-ranging impact on circuit function (Csicsvari et al., 1998; Fujisawa et al., 2008; Gulyás et al., 1993; Marshall et al., 2002; Maurer et al., 2006; Miles, 1990).

In this study, we examined whether old and newly established network assemblies flicker to test the hypothesis that hippocampal map competition occurs during spatial learning. In addition, we investigated the contribution of inhibitory circuits by testing the hypothesis that the formation of behaviorally-relevant pyramidal cell assemblies involves the modification of inhibitory microcircuits. We found that the flickering of old and new maps takes place during spatial learning. Surprisingly, many interneurons reorganized their firing patterns during learning, forming dynamic associations to the new assemblies in relation to the assembly flickering. Moreover, by measuring spike transmission probability between monosynaptic pyramidal cell-interneuron pairs, we assessed changes of local excitatory connections onto these interneurons. We found that pyramidal cell connections to interneurons exhibited map-specific changes that were developed during learning, which in turn can explain the newly formed associations between interneuron firing and pyramidal assemblies.

## Results

To explore how interneurons change their coupling strength to pyramidal cell assemblies during spatial learning, hippocampus circuit activity from the CA1 pyramidal cell layer was recorded using multichannel extracellular techniques in rats performing a spatial learning task on a cheeseboard maze (see Figure S1 available online; Experimental Procedures; Dupret et al., 2010). Some of the data used here were collected for the previous Dupret et al. (2010) study (seven animals). In this task, animals learned the locations of three new goals where food reward were hidden each day. The animal’s memory performance was assessed before and after the learning (preprobe and the postprobe sessions) and the animals were allowed to sleep before and after the learning in presleep and postsleep sessions (Figure S1). During learning some of the place cells remapped their place fields. Moreover, the successful recall of newly learned goal locations in the postprobe session was associated with the reinstatement of the new place field representations that were developed during learning (Dupret et al., 2010).

### Firing Association of Interneurons to Pyramidal Cell Assemblies

**Figure 1:**
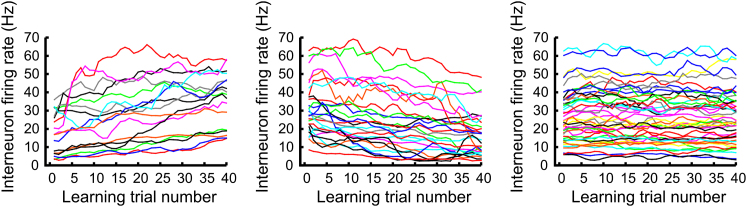
Interneuron Mean Firing Rate during Spatial Learning Examples of CA1 hippocampal interneuron firing rate (Hz) time course during 40 consecutive learning trials. The mean firing rate of many individual interneurons was altered during the course of learning, either increasing (left) or decreasing (middle), while the firing rate of others remained stable (right). See also Figures S1 and S2.

First, we examined whether spatial learning was accompanied by interneuron firing rate changes as reported during exploration of novel environments (Frank et al., 2004; Nitz and McNaughton, 2004; Wilson and McNaughton, 1993). Firing rate changes of interneurons were observed during learning on the cheeseboard maze, and these followed a similar time course to the reorganization of pyramidal cell assemblies. About 25% of interneurons exhibited significant increases in their rate, while an additional 43% showed significant decreases (Figure 1). Such mean rate changes of interneurons were not observed when the animals performed the task without the allocentric learning context where reward locations were indicated by intramaze cues (Figure S2). Since the behavioral patterns of the animals during the cued and the allocentric conditions were similar, it is unlikely that interneuron rate changes were attributed to behavioral changes or related factors such as the speed of the animal. Instead, the observed interneuron rate changes might have signaled the formation of new associations to new pyramidal assemblies that were developed during the allocentric learning of reward locations.

**Figure 2:**
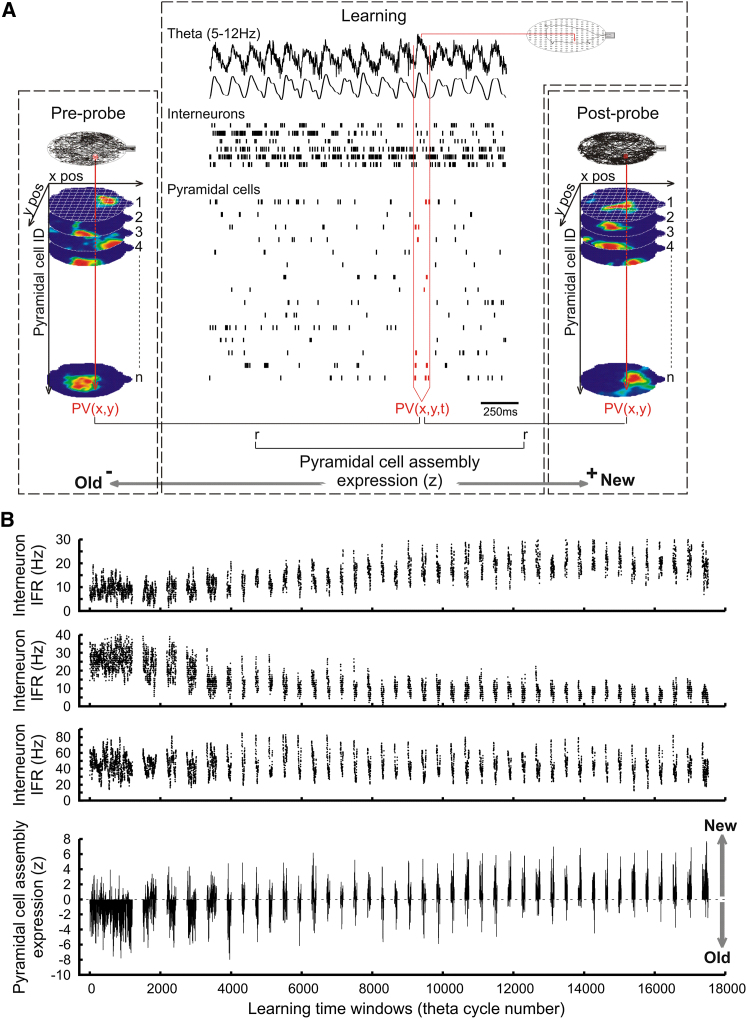
Temporal Fluctuations of the Interneurons Rate and the Expression of Pyramidal Cell Maps during Learning (A) Procedure for analyzing interneurons and pyramidal cell assemblies’ firing dynamics. Theta cycles in each learning trials were used as time windows to both calculate the instantaneous firing rate (“IFR”) of interneurons and to identify the ongoing hippocampal maps expressed by pyramidal assemblies using a population vector-based analysis. First the rate maps of CA1 pyramidal cells were stacked into three-dimensional matrices for both probe sessions preceding and following learning (the two spatial dimensions on the x and y axis, cell identity on the z axis); thus each x-y pixel was represented by a population vector composed by the pyramidal cell firing rate at that location. Next in each theta cycle, the instantaneous spike counts of the pyramidal cells were used to establish a population vector; each of these ongoing vectors was correlated with the corresponding x-y vector from both the preprobe and the postprobe (“r”) and the correlation coefficients compared with a Fisher z test (“z”). Positive z values indicate times when pyramidal firing patterns preferentially expressed the new cell assemblies (“New”) while negative values indicate the expression of the old ones (“Old”). See also Experimental Procedures and Figure S3. (B) Examples of instantaneous firing rate (Hz) of individual interneurons and pyramidal cell assembly expression values (z) during a learning session. Each block represents a trial spaced by intertrial intervals.

To test for the development of interneuron associations to new pyramidal assemblies, we examined whether interneuron rates mirrored the dynamic reorganization of pyramidal assemblies during map formation. High-fidelity associations would require interneurons to fire stronger in time periods when new maps are accurately expressed. In contrast, a negative association may signal that interneurons reduce their firing when the newly formed pyramidal patterns are present. Pyramidal cell assemblies can rapidly switch across theta cycles when certain environmental features are rapidly altered (Jezek et al., 2011). In our analysis we also used theta cycles (5–12 Hz) as time windows to measure the instantaneous firing rate of interneurons and to quantify the firing association of interneurons to pyramidal assembly patterns (Figure 2). The expression of the new maps was assessed in each theta cycle by testing whether the ongoing pyramidal network activity was more similar to the old or the new assembly patterns representing the current location. Hence, during learning, the instantaneous firing rates of all recorded pyramidal cells were correlated to population vectors taken from place maps expressed in the preprobe and postprobe sessions (Figures 2 and S3A–S3D). Comparing these two correlations provided a measure determining which assembly (i.e., old or new) has been expressed in a given theta cycle during learning (see Experimental Procedures). Positive assembly expression values indicate times at which the pyramidal activity patterns preferentially expressed the new cell assemblies during learning (i.e., more similar to the postprobe), while negative ones point to the expression of the old assemblies (i.e., more similar to the preprobe). The instantaneous assembly expression values indicated that within many earlier trials, both the old and the new pyramidal assembly representations were expressed in nonoverlapping theta cycles, with later trials dominated by the new patterns (Figures 2 and S3A–S3D). Moreover, the expression strength of the new assemblies improved during the course of learning, suggesting their refinement. Similar expression of the new and old assemblies can be observed when measured within gamma oscillatory cycles (30–80 Hz; see Experimental Procedures), and the assembly expression scores measured during gamma oscillations correlated significantly (p < 0.00001) with those measured in the overlaying theta cycles (Figures S3E–S3G). These temporal fluctuations between distinct assemblies were not merely resulting from a change in the animal’s trajectory as no such reorganization of place cell assemblies occurred in the cued version of the task (Dupret et al., 2010). The switching between old and new assemblies observed here is similar to previous studies in which cell assembly patterns rapidly flicker between distinct representations of the same location (Jackson and Redish, 2007; Jezek et al., 2011; Kelemen and Fenton, 2010).

**Figure 3:**
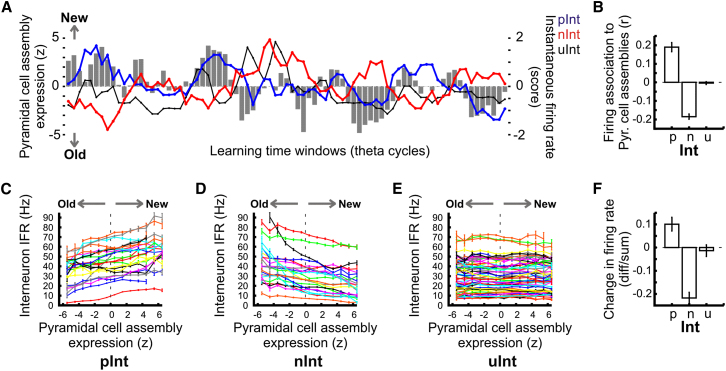
Interneuron Firing Associations to Pyramidal Cell Assemblies during Learning (A) Expanded trace showing the rapid rate fluctuation of three simultaneously recorded interneurons from different groups (colored lines: blue, pInt; red, nInt; black, uInt) with the expression of pyramidal cell assemblies (gray impulses) over consecutive theta cycles. Interneuron instantaneous firing rate scores are used to display rapid fluctuations around the mean. Positive z values for pyramidal assembly expression indicate times when the new maps were expressed while the negative ones indicate times when the old maps revert back. (B–E) Firing association of the different interneuron groups to pyramidal assemblies. During learning, the IFR of many interneurons was either positively (“pInt”) or negatively (“nInt”) correlated with the pyramidal assembly expression (B), mean ± SEM, all p’s < 0.0001) while others were uncorrelated (B), “uInt,” p = 0.794). (F) Change in firing rate of different interneuron groups (mean ± SEM) measured as the rate difference between the first and the last 10 min of learning divided by the sum. The pInt and the nInt interneurons exhibited significant changes (all p’s < 0.0014) but not the uInt group (“u,” p = 0.846). See also Figures S1, S2, S4, and S5.

The firing rate of many interneurons also fluctuated on a fast time scale that followed this assembly flickering (Figure 3A). As suggested by data from the cued task, these rate fluctuations of interneurons associated with allocentric learning were bigger than those that could be expected due to changes in locomotor, spatial behavior or by natural intrinsic variability (Figures S2D and S2E). Moreover, 72% of our CA1 interneurons exhibited a significant correlation (p < 0.05) between their instantaneous firing rate and the theta-paced expression strength of new pyramidal assemblies. Those that exhibited significant positive correlations—referred to as “*pInt*” – increased their instantaneous rate at times when the new representation was preferentially expressed (Figures 3B and 3C; n = 86 interneurons) while the ones with negative correlation – referred to as “*nInt*”—decreased their firing during the same moments (Figures 3B and 3D; n = 131 interneurons). These firing associations of interneurons to pyramidal assemblies were measured at the end of learning (Figure 3B; last 10 trials) to minimize possible biases due to the gradual drift of interneuron firing rate in the establishment of assembly associations. The observed interneuron activities were inherently driven by associations to entire hippocampal maps, and not merely to assemblies bound to a particular position of the animal, nor explained by other learning-independent behavioral parameters such as the speed of the animal (Figure S4). As the new pyramidal representations occurred more often than the old ones toward the later trials, the *pInt* and *nInt* interneuron groups increased and decreased their mean firing rate during the course of learning respectively (Figure 3F); however, these rate changes were restricted to the learning period (Figure S1D). Therefore, the cell assembly associations of interneuron measured at the end of learning predicted rate changes of interneurons during the whole course of learning. This suggests that the observed rate changes occurred as a consequence of the development of association to pyramidal assemblies. Note that 28% of interneurons did not show significant associational changes with the expression of pyramidal assemblies (referred to as “uInt”; Figures 3B and 3E; n = 85 interneuron) and exhibited stable firing rates (Figures 3F and S1D) during the course of learning.

Interestingly, pInt and nInt interneurons exhibited overlapping but significantly different distributions of their preferred theta phase (p < 0.024, Watson-Williams test) and a tendency toward a difference in strength of gamma phase locking (p = 0.095), demonstrating that these two cell groups exhibited physiological differences beyond their association to pyramidal assemblies (Figure S5).

### Changes in Pyramidal Cell-Interneuron Connection Strength

**Figure 4:**
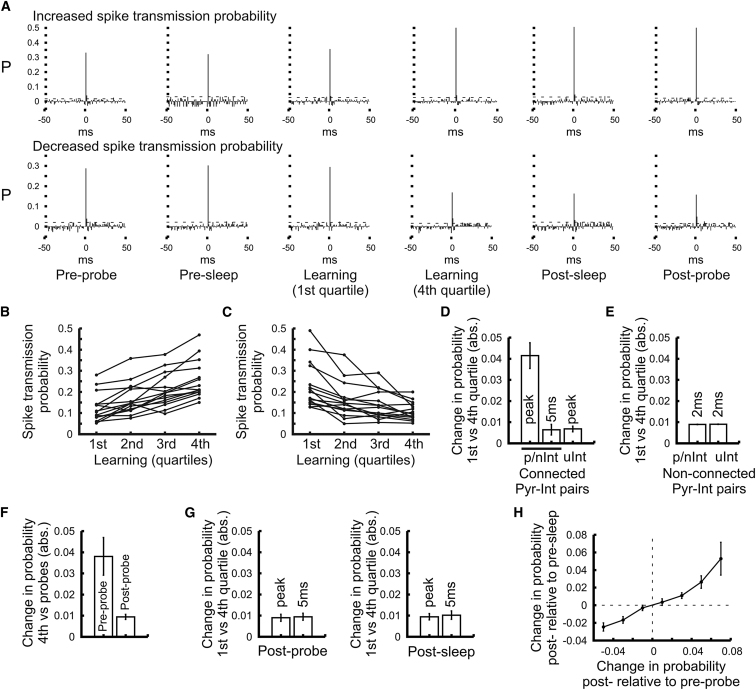
Learning-Related Changes in Pyramidal Cell-Interneuron Coupling Strength (A) Examples of cross-correlograms calculated for two pyramidal cell-interneuron pairs (one row each pair) in behavioral sessions performed sequentially by the animal (the first and the fourth quartiles are depicted for the learning). In these cross-correlograms the pyramidal cell action potentials were used as reference and the joint firing probability that the two cells fire together by chance (calculated from the first and last 20 ms bins) was subtracted in order to account for firing rate change-related fluctuations in the correlation strength. Dashed lines represent 3SD from the mean. Note the presence of a large, sharp peak at short-latency (<3 ms) as the signature of an excitatory monosynaptic connection and that the spike transmission probability can either increase (top row) or decrease (bottom row) after learning. See also Figure S6. (B and C) Learning-related changes in pyramidal cell-interneuron spike transmission probability. The transmission probability was calculated for learning quartiles and displayed for pairs which spike transmission either increase (B) or decrease (C) during learning; 15 examples are depicted for each case. (D and E) Absolute change in transmission probability (mean ± SEM) during learning (first versus fourth quartile). Cell pairs of pyramidal cells connected (D) or not (E) to interneurons of the different groups (p/nInt and uInt) were identified respectively from the presence or not of a significant peak at monosynaptic latency (<3 ms) in the cross-correlograms. The probabilities were calculated at the monosynaptic latency (peak and 2 ms bins for connected and nonconnected pairs, respectively). For connected pairs changes were also measured at 5 ms bin. (F and G) Absolute change in spike transmission probability for monosynaptically connected pyramidal cell to p/nInt interneurons (mean ± SEM). (F) The change was calculated between the end of the learning and the probe sessions (fourth quartile versus Preprobe or Postprobe). (G) The change within the probe and the sleep sessions (first versus fourth quartile) following learning was calculated at the monosynaptic peak and at the 5ms bin. (H) Change in spike transmission probability across the sleep sessions as a function of the change in transmission probability across probe sessions (mean ± SEM). See also Figures S2 and S5–S8.

The firing association of interneurons to pyramidal assemblies may have taken place because interneurons had changed the connection strength with their presynaptic pyramidal cells. Had such learning-related connection changes taken place, these were expected to develop during the learning without further alterations in the subsequent postprobe session. Monosynaptically connected pyramidal cell-interneuron pairs were identified by the presence of a sharp peak at short latency (<3 ms after the discharge of the reference pyramidal cell) in the pyramidal cell-interneuron cross-correlation histograms (Figure S6A; mean peak probability: 0.101 ± 0.006, maximum 0.521; mean peak latency: 1.546 ± 0.038 ms) (Csicsvari et al., 1998; Fujisawa et al., 2008; Marshall et al., 2002; Maurer et al., 2006). The connection strength was thus accessed by measuring the spike transmission probability at the monosynaptic peak bins (i.e., 0.5–2.5 ms). However, the firing probability that the two cells fire together by chance at nearby 30–50 ms bins in both sides of the histograms was subtracted from the correlation strength in order to remove possible changes in the joint firing probability caused by local rate changes. In many instances the spike transmission probability between a given pyramidal cell and its target interneuron either decreased (n = 126 pairs) or increased (n = 98 pairs) after learning, as shown by the comparison of the cross-correlograms calculated for sessions before and after learning (Figures 4A and S6B). Such changes in pyramidal cell-interneuron transmission probability developed during learning (Figures 4B and 4C). Moreover, these learning-related weight changes did not exhibit further changes after learning: the transmission probability observed at the end of learning remained stable in the following postprobe session with no further changes during sleep or probe sessions (Figures 4F and 4G). The observed changes in spike transmission to p/nInt interneurons occurred during the monosynaptic delay period (0.5–2.5 ms) only, and did not affect bins outside this delay at the 5ms bins (Figure 4D) or at the 30–50 ms bins. The changes in absolute value of the transmission probability were much smaller for the 5 ms or the 30–50 ms bins as compared to the monosynaptic bins (first versus fourth learning quartile; 30–50 ms bin: 0.0084 ± 0.0009, 5 ms bin: 0.0071 ± 0.0019; p = 0.623) and not correlated with those at the monosynaptic bins (0.5–2.5 ms; p = 0.549) nor with those at the 5ms bins (p = 0.626). Similar results were found with pyramidal cell-interneuron cross-correlograms by measuring the correlation coefficients of spike coincidence, which measure is independent of the firing rate of both cells (Figures S6C–S6F). Moreover, other cell pairs that did not exhibit significant monosynaptic peaks did not show such changes in transmission probability at the 2 ms monosynaptic latency bin, even though these cells underwent similar spatial changes in firing rate (Figure 4E; n = 14522 pairs). Had local (spatial) changes in firing rate been the cause of the correlation changes of the monosynaptic pairs, they should have equally influenced bins at 5 ms or other cell pairs at 2 ms in which monosynaptic peaks were not detected. Thus, the observed changes in spike transmission probability could not be explained by changes in place-related firing of pyramidal cell and/or interneurons or by the firing associations we measured between them. These factors would have affected joint firing across longer time delays and not solely at monosynaptic latencies, and they would have also influenced correlations in which the monosynaptic connection has not been detected. It is unlikely that learning-related changes in spike transmission probability were caused by theta phase-related changes as pyramidal cell-interneurons cross-correlograms did not exhibit visible theta modulation (Figures 4A and S6) and changes in theta firing preference of both interneurons and pyramidal cells were not related to changes in spike transmission probability (Figures S7 and S8). Inherently these changes were linked to spatial learning as no such learning-related changes in the coupling strength were observed in the intra-maze cued task (Figure S2F).

**Figure 5:**
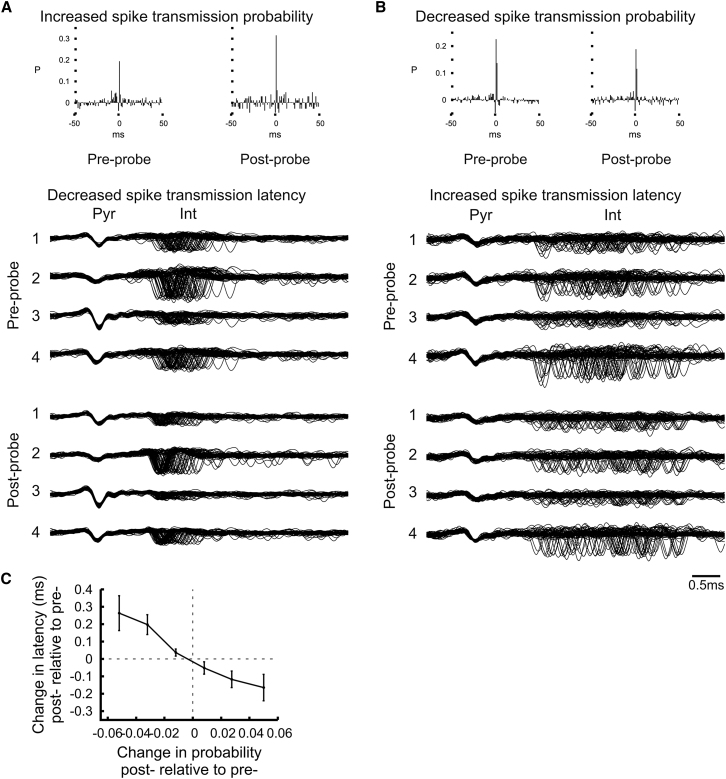
Changes in Pyramidal Cell-Interneuron Spike Transmission Latency (A and B) Examples showing that strengthened (A) and weakened (B) pyramidal cell-interneuron connections are accompanied by reduction and increase of spike transmission latency respectively. The multiple sweeps show expanded superimposed waveforms of a pyramidal cell (“Pyr”) and its target interneuron (“Int”) from the preprobe and the postprobe sessions that were recorded from the same tetrode. All four channels of the tetrode are plotted. Note that the interneurons often fired at a short (<3 ms) but variable latency after their presynaptic pyramidal cell. Note the left shift toward shorter pyramidal cell-interneuron latencies associated with increased transmission probability after learning (A) but the right shift toward longer latencies in the case of decreased transmission probability (B). (C) Change in spike transmission latency as a function of the change in transmission probability (postprobe relative to preprobe, mean ± SEM). Note that the stronger was the spike transmission probability, the faster it was. See also Figures S5, S7, and S8.

The changes in spike transmission probability observed across the probe sessions before and after learning were correlated with those measured across the sleep sessions (Figure 4H; r = 0.549, p < 0.00001; Figure S6E), suggesting that they reflected enduring modification of the excitatory synaptic drive onto these interneurons rather than behavioral state-dependent modulatory mechanisms. Moreover, these learning-related changes in transmission probability were also accompanied by changes of spike transmission latency (Figures 5A and 5B; mean change of latency ± SEM: for increased probability pairs = –0.228 ± 0.08 ms, for decreased probability pairs = 0.232 ± 0.103 ms). Indeed, the stronger the transmission probability after learning, the faster the spike transmission (Figure 5C; r = –0.346, p < 0.00001) and changes in transmission latency observed across the probe sessions before and after learning correlated with those across sleep sessions (r = 0.326, p < 0.0007). These changes in spike transmission latency suggest plastic changes as faster and slower rise times of excitatory postsynaptic potentials have been associated with the facilitation and depression of pyramidal cell-interneuron synapses respectively (Alle et al., 2001; Lamsa et al., 2005, 2007; Perez et al., 2001). As for the changes in transmission probability, such short changes in spike transmission latency cannot be explained by local firing rate changes of pyramidal cells and interneurons during learning.

### Assembly Membership-Related Modification of Pyramidal Cell-Interneuron Connection

**Figure 6:**
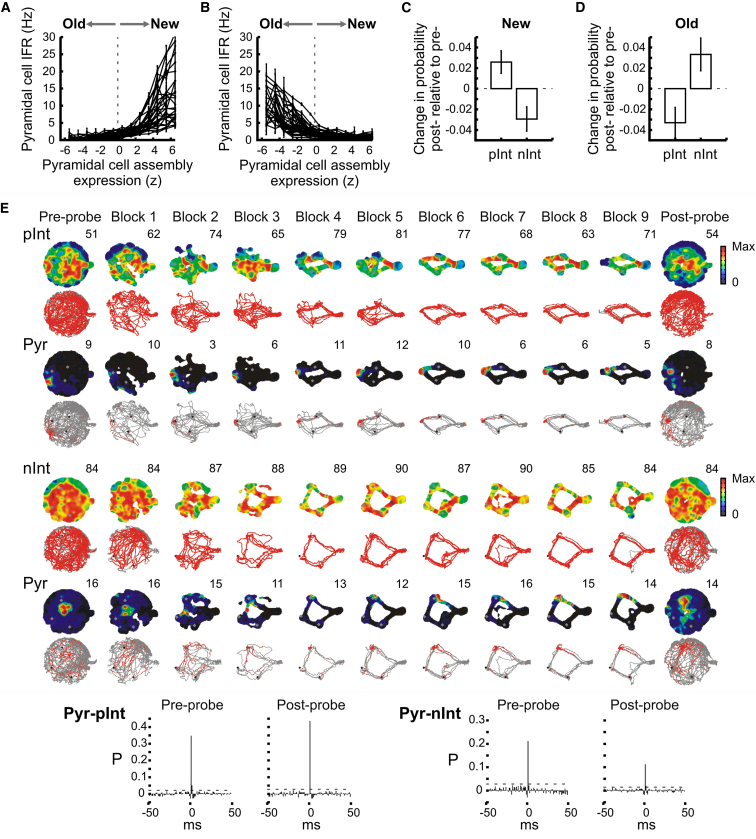
Assembly Membership Dependency of Pyramidal Cell-Interneuron Connection Changes (A and B) Assembly membership of individual pyramidal cells as established by the correlation between the instantaneous firing rate of the pyramidal cell (Hz) and the assembly expression (z). During learning, those pyramidal cells that exhibited a significant positive correlation were assigned as members of the new cell assembly (A) whereas those that showed significant negative correlation were part of the old assembly (B). (C and D) Change in pyramidal-interneuron spike transmission probability (mean ± SEM) from the preprobe to the postprobe according to the pyramidal cell assembly membership. Pyramidal cells that were members of a new assembly strengthened their connections to pInt interneurons while weakened their connections to nInt interneurons (C, all p’s < 0.025); the opposite changes were observed with pyramidal cells linked to the old assemblies (D, all p’s < 0.036). Thus pyramidal cells and interneurons that are members of a newly formed assembly strengthened their connections. (E) Examples of a pInt (top) and a nInt (bottom) interneuron each simultaneously recorded with a place cell (“Pyr”) that exhibited a firing field at one of the goal locations and thus was part of a new assembly. Alternating rows showed color-coded place rate maps (with peak rate values indicated) and individual spike locations (red dots) superimposed on the animal’s path (gray traces) for the probe sessions and the consecutive blocks of learning trials. Gray and black dots indicate goal locations. The cross-correlograms for these pairs are shown. See also Figures S7 and S8.

It is possible that the changes of connection weight we observed between pyramidal cells and interneurons contributed to the firing associations we observed between them. If this is the case, we expect that *pInt* interneurons strengthened their connections with pyramidal cells that were part of a new assembly, and reduced those with pyramidal cells of an old assembly. Conversely, we would expect the opposite changes for *nInt* interneurons. To identify pyramidal cells that were part of a new assembly, we identified those that preferentially fired when the new assemblies were expressed as compared to the old ones (Figures 6A and 6B; see Experimental Procedures). That is, we selected cells whose instantaneous firing rate correlated positively with the expression strength of the new pyramidal assemblies in last 10 learning trials (mean r = 0.116 ± 0.003, n = 996). However, pyramidal cells that preferentially fired with the old maps had a negative correlation with the assemblies expression score (mean r = –0.102 ± 0.002, n = 101). Importantly pyramidal cells that were members of a new assembly strengthened their connections with the *pInt* interneurons while the same pyramidal cells decreased their connections to the *nInt* interneurons (Figures 6C and S6G; all p’s < 0.030). The opposite changes were observed with pyramidal cells that were linked to the old assemblies (Figures 6D and S6H; all p’s < 0.036). These changes in connections all promote an increase of associations for *pInt* interneurons to the new assemblies and the decoupling of *nInt* interneurons to the same assemblies.

The analysis above considered only those pyramidal cells that preferentially fired at times when either the old or the new maps were present during learning. This type of analysis however excluded those pyramidal cells that were active both with the old and the new cell assemblies. Therefore, in a further analysis we used new assembly-associated firing rate as a predictor of membership. We also reasoned that for interneurons to accurately associate or dissociate with the expression of the new maps, the changes in connection strength with their presynaptic pyramidal cells should reflect the strength by which the pyramidal cell is active when participating in the new assembly firing. Indeed the stronger the presynaptic pyramidal cells fire at times when the new assemblies were expressed during learning, the stronger the increase in their connection strength with *pInt* interneurons was across probe sessions (r = 0.367, p = 0.030); the opposite relationship was observed with the *nInt* interneurons (r = –0.430, p = 0.012). In this analysis normalized firing rate were correlated with the change in spike transmission probability.

Finally, we used a complementary analysis based on place field remapping to select pyramidal cells that became part of a new assembly. We selected those pyramidal cells that remapped their place fields between the probe sessions before and after learning and exhibited fine spatial tuning in the postprobe session (place field similarity < 0.2, sparsity < 0.3; coherence > 0.6; see Experimental Procedures). Next, we calculated the average change in spike transmission probability of these place cells with the *pInt* and the *nInt* interneurons across the probe sessions (see examples in Figure 6E). Pyramidal cells that remapped their place fields exhibited a significant increase of spike transmission probability with *pInt* interneurons but a significant reduction with *nInt* interneurons (*pInt* = 0.040 ± 0.019, n = 31 pairs; *nInt* = –0.038 ± 0.012, n = 54 pairs; all p’s < 0.042).

### Contribution of the Pyramidal Cell-Interneuron Pairing Activity

**Figure 7:**
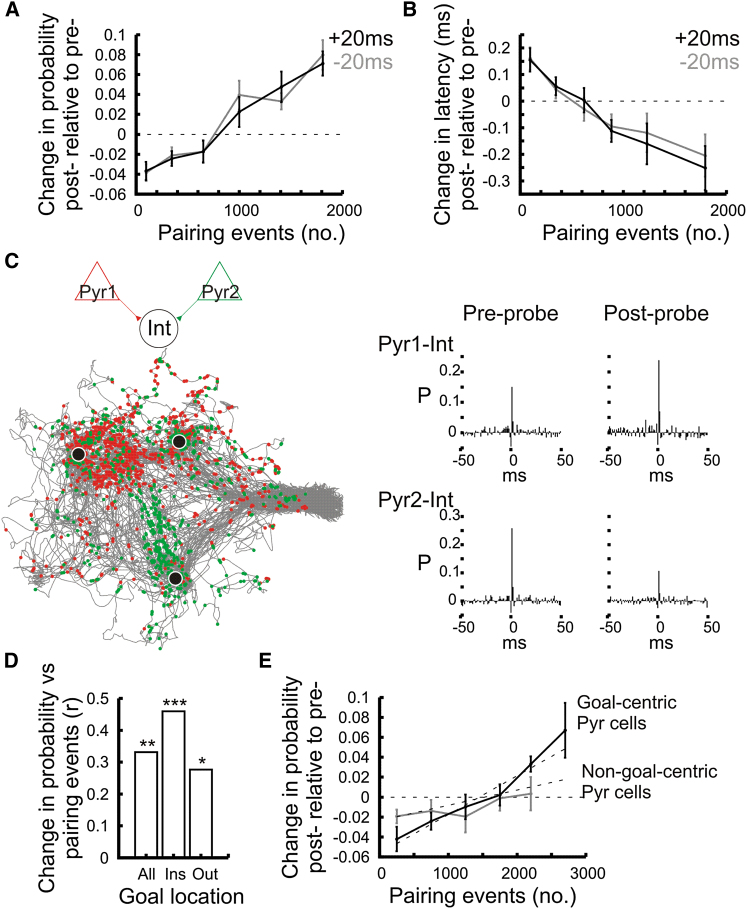
Pyramidal Cell-Interneuron Pairing Activity during Learning (A and B) Change in spike transmission probability (A) and latency (B) from the preprobe to the postprobe as a function of the number of pairing events during learning (mean ± SEM). Pairing events refer to the number of 20 ms time windows in which pyramidal cell spikes were preceded (gray curves) or followed (black curves) by interneuron spikes. (C) Spatial location of pairing events. Color dots mark the locations of the pairing events between two pyramidal cells and the same interneuron (see cross-correlation on the right) superimposed on the animal movement path during learning (in gray). Black dots indicate goal locations. Note that pairing events occurred both inside and outside goal locations. (D) Correlation coefficients between the number of pairing events during learning and the change in transmission probability from the preprobe to the postprobe session. “All” = all events; “Ins” = events inside goal areas; “Out” = events outside goal areas. (E) Change in spike transmission probability (post- relative to preprobe, mean ± SEM) as a function of the number of pairing events during learning for pyramidal-cell-interneuron cell pairs involving those pyramidal cells that exhibited goal-centric firing and those that did not. See also Figures S7 and S8.

Collectively, the above results demonstrate that *pInt* interneurons specifically increased their connection strength with those pyramidal cells that were part of the new assemblies, while a decreased connection was observed for *nInt* interneurons. These connection changes facilitated the assembly-related association of interneuron firing. Further, we aimed to identify factors that may have led to the connection changes promoting the cell assembly-specific firing association of interneurons. Since active pyramidal cells can both strengthen or weaken their connection with their postsynaptic interneuron partners (Figure 6E), we reasoned that the pairing of the interneuron and the pyramidal cell firing may be a factor that predicts connection change. First, we examined whether pyramidal cell-interneuron connection changes could be predicted by the number of pairing events (calculated during theta epochs in learning) during which the pyramidal cell firing was preceded or followed by interneuron action potentials within 20 ms. Indeed, the change in spike transmission probability between probe sessions correlated with the number of pairing events during learning, independent of whether the interneuron fired before or after the pyramidal cell (Figure 7A; −20 ms: r = 0.394; +20 ms: r = 0.398; all p’s < 0.00001). This was the case for both the *nInt* (r = 0.222, p = 0.026) and the *pInt* (r = 0.419; p = 0.013). Moreover, the number of pairing events was also associated with a change in transmission latency: the more often pyramidal cells were paired with an interneurons spike during learning, the shorter the subsequent pyramidal cell-interneuron connection delay (Figure 7B; –20 ms: r = 0.432; +20 ms: r = 0.442; all p’s < 0.00001).

We showed above that the number of pairing events predicted the change of pyramidal cell-interneuron connection changes. However, the number of pairings with pyramidal cells during learning does not guarantee that specific associations are made with newly formed assemblies, since old assemblies are also intermittently present during learning trials. Because the reorganization of place cells were focused on newly learned goal locations, pairing events at these locations may have been more efficient at shaping the connections. Thus, we determined whether neuronal pairing at goal locations facilitated the strengthening or weakening of synaptic connections. Spike-pairing events (±20 ms time difference) occurred both inside and outside the goal areas (Figure 7C) although more occurred outside than inside (inside = 133.8 ± 16.7, outside = 850.4 ± 65.1, p < 0.00001, t test). Nevertheless, the change in transmission probability was better predicted by pairings occurring inside goal areas (Figure 7D). Consistent with this, the strengthening of the pyramidal cell-interneuron connection was greater when the pre-synaptic pyramidal cell exhibited goal-centric firing (Figure 7E; goal-centric cells: r = 0.581; non-goal-centric cells: r = 0.232; Z = 2.163, Fisher z-test), as indicated by a steeper slope of the regression line (goal-centric cells > non-goal-centric cells, p = 0.010). Together these results suggest that the coincident firing of the pyramidal cells and their target interneurons governed changes of their connection strength and that such pairing was more effective in influencing connection changes when it took place at the newly learned goal locations.

### Contribution of the Coincident Interneuron Activity State

**Figure 8:**
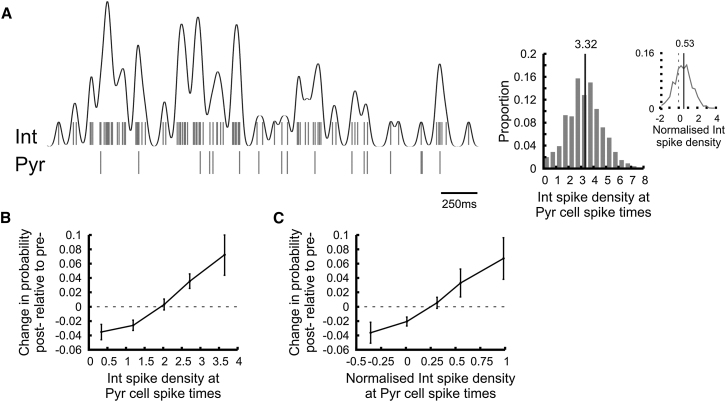
Interneuron Excitation State at Pyramidal Cell Spike Times (A) An example of interneuron spike density and coincident pyramidal cell firing. The spike train of the interneuron (shown as a raster plot) was convolved with a Gaussian kernel (SD = 20 ms) to provide a continuous measure of its excitation state (black curve). The histograms on the right show the distribution of the interneuron spike density at pyramidal cell spike times (raw values and normalized value). The mean value is marked by vertical black lines. (B and C) Change in transmission probability across probe sessions as a function of the mean interneuron spike density at pyramidal cell spike times (B): raw density values; (C): density normalized by the mean firing rate).

In vitro experiments have suggested that some postsynaptic interneurons need to be depolarized to observe synaptic changes, suggesting that the ongoing interneuron excitation state can influence pyramidal cell-interneuron connection changes. Spike trains of interneurons were convolved with a one-dimensional Gaussian kernel with a width parameter σ of 20 ms to provide a continuous measure of their spike density during learning (Figure 8A; Kruskal et al., 2007). We found that the change in transmission probability measured across the probe sessions positively correlated with the mean interneuron spike density measured during learning at times when the presynaptic pyramidal cell fired an action potential (Figure 8B; r = 0.405, p < 0.00001). This correlation remained significant even when the ongoing spike density was controlled by the mean interneuron firing rate (Figure 8C; r = 0.375, p < 0.00001). Moreover, the contribution of the coincident interneuron depolarization state to the change in the transmission probability was still significant when controlled for the total number of pyramidal cell-interneuron 20ms pairing events (r = 0.268, p = 0.0008, partial correlation) and for running speed at times of the spike coincident events (r = 0.280, p = 0.0022, partial correlation). These results showed that temporal coincidence between the pre-synaptic pyramidal cell spikes and the postsynaptic interneuron excitation state further contributed to the direction and the magnitude of the synaptic changes.

## Discussion

In this study, we have shown that spatial learning on the cheeseboard maze was associated with the dynamic reconfiguration of interneuron circuits in the CA1 pyramidal cell layer of the hippocampus. The strength of the local input that interneurons received from pyramidal cells was altered during learning, and, as a result, many of them developed firing associations to newly formed pyramidal assemblies that were part of the spatial maps representing information about recently acquired spatial memories. While the firing of some interneurons was bound to the expression of new pyramidal assemblies, other interneurons dissociated their firing from the activity of the same assemblies. These firing associations, manifested by rapid fluctuations of the interneurons firing rate, were mirrored by changes of their monosynaptic connection weight. Interneurons that increased their firing associations to new pyramidal assemblies overall received strengthened inputs from pyramidal cells that were members of a new assembly. Moreover, the opposite trend was observed for interneurons that decreased their associations to new assemblies, these received weaker local pyramidal inputs following learning. Importantly, this circuit reconfiguration took place during the learning session and it remained stable in subsequent sleep and memory probe sessions.

In analyzing the temporal expression of pyramidal assemblies representing old and newly developed maps during learning, we found that the old assemblies were present even later during learning, with old and new cell assemblies alternating even within a single learning trial. In addition, assemblies of the new maps emerged rather abruptly, in parallel with the rapid improvement of the behavioral performance of the animal within the initial learning trials. As learning progressed the newly established maps were then refined, together with an increase of the frequency of the new assemblies, and thus dominated late learning periods. The rapid formation of new hippocampal maps is consistently observed when an animal is first placed in a novel environment (Frank et al., 2004; Leutgeb et al., 2004; Wilson and McNaughton, 1993). In this study, the formation of new maps took place during goal-directed spatial learning in an otherwise familiar environment. Map formation may still share similar processes to those of forming spatial representations of new environments; albeit in this latter case map refinement has been observed on a slower time scale, over consecutive days in the CA1 region (Frank et al., 2004; Lever et al., 2002). Similar rapid assembly flickering between competing maps has also been observed in cases where the animal remained in the same environment but the task contingencies or some environmental features were suddenly changed (Jackson and Redish, 2007; Jezek et al., 2011; Kelemen and Fenton, 2010). Here, we further show that rapid flickering of pyramidal assemblies took place during spatial learning of new goal locations in the same environment with the same spatial cues being present. The fact that the old map recurs throughout learning in our behavioral paradigm suggests that the animal retains information about the old map as it is uncertain whether the change of reward locations was transient or long-lasting. This is consistent with a previous study showing that the coordination of multiple spatial maps is needed to prevent confusion and select the appropriate behavioral response during a two-frame place avoidance task (Kelemen and Fenton, 2010). Thus, the observed map switching in our study suggests a competitive process in which the newly formed map gains influence as it can successfully predict current goal locations needed for the animal to solve the task. However, the mechanisms by which behaviorally relevant maps are selected from the flickering alternatives to guide behavior is yet to be resolved to establish a closer link between cell assembly flickering and behavioral performance.

Interestingly, theta-paced flickering of pyramidal cell assemblies we observed also extended to the gamma timescale. We show that pyramidal assembly expression scores measured during gamma oscillations correlated with those measured in corresponding theta oscillatory cycles. These results might indicate the existence of a dual coding scheme where theta-paced assembly flickering determines which maps are present while gamma oscillations may code for sequences of visited places of a movement path (Lisman, 2005).

A change of interneuron firing rate has been previously reported during exploration of novel environments (Frank et al., 2004; Nitz and McNaughton, 2004; Wilson and McNaughton, 1993). We have observed separate populations of interneurons that either increased or decreased their firing rate within spatial learning sessions. However, in our paradigm rate modulation was stronger than that observed in a novel environment (2- to 3-fold change in some cases), suggesting that interneurons rate association is stronger in goal-associated learning. Importantly, the direction of firing rate changes was predicted by the firing associations of interneurons to pyramidal assemblies.

Overall, our data suggest that interneurons specifically changed the input connections from newly formed pyramidal assemblies representing the new map. Given that interneurons receive inputs from many presynaptic CA1 pyramidal cells (Ali et al., 1998; Freund and Buzsáki, 1996; Gulyás et al., 1993), this enables them to integrate the activity of those that belong to assemblies of the new map. Therefore, interneurons can accurately code for the expression strength of new cell assemblies by the rapid fluctuations of their firing rates. This in turn enables the dynamic regulation of excitability in hippocampal subcircuits, depending on the expression strength of assemblies. Such regulation of excitability could facilitate neuronal plasticity in time periods when new assemblies were accurately expressed. In this way, the enhanced inhibition provided by *pInt* interneurons can facilitate the temporal synchronization of pyramidal cells leading to more favorable conditions to alter pyramidal-pyramidal connections. In contrast, inhibition provided by *nInt* interneurons is reduced at the same time, which could facilitate calcium entry or even regulate the formation of dendritic calcium spikes (Klausberger, 2009; Miles et al., 1996; Pouille and Scanziani, 2004). Future work may allow to test whether pInt and nInt interneurons, both recorded in the pyramidal cell layer, correspond with different interneuron types (Klausberger and Somogyi, 2008; Somogyi and Klausberger, 2005), considering advances in identifying cell categories in multichannel recorded data (Czurkó et al., 2011) and those enabling juxtacellularly recorded/labeling in freely moving rats (Lapray et al., 2012). The regulation of plasticity would be favorable during awake sharp wave/ripple (SWR) events that occurred at reward locations (Dupret et al., 2010; Singer and Frank, 2009). During such network events, place cells have been found to enhance their ongoing place-selective activity, which could provide the conditions for the online strengthening of newly formed maps (Carr et al., 2011; Dupret et al., 2010; O’Neill et al., 2010; Singer and Frank, 2009).

In the scenarios above, we suggested that interneuron firing rate modulation may promote assembly stabilization by regulating plasticity within pyramidal cell assemblies. Plasticity at pyramidal cell-interneuron synapses may thus help to improve the signal-to-noise ratio of assembly expression and contribute to processes that maintain the integrity of maps. In such a case, different combinations of interneurons are associated with different pyramidal maps, and, as such, contribute to the segregation of pyramidal activity coding different maps (Buzsáki, 2010).

In this work, we have been able to provide a mechanistic explanation for the association of interneurons to pyramidal assemblies. To do so, we estimated changes of connection weights from CA1 pyramidal cells to interneurons in vivo by measuring the spike transmission probability between cell pairs with cross-correlograms pointing to monosynaptic connections. These changes were observed at the monosynaptic delay period only and for those pyramidal cell-interneuron pairs that were monosynaptically coupled. Hence the observed monosynaptic changes were not caused by spurious probability changes caused by the measured association of interneurons to pyramidal assemblies. Moreover neuromodulatory changes that might cause changes of interneurons membrane potential cannot explain monosynaptic transmission changes either, as the changes were observed only during learning and maintained subsequently in waking probe and sleep sessions. Therefore, these findings all suggest that synaptic connection weight changes between pyramidal cells and interneurons are a cause of the cell assembly associations. In demonstrating these correlation changes, we have been able to provide evidence for the dynamic reconfiguration of interneuron circuits in relation to spatial learning. This is consistent with in vitro studies that have demonstrated that glutamatergic synapses from excitatory principal cells onto GABAergic interneurons in the hippocampus are modifiable in an activity-dependent manner (Alle et al., 2001; Lamsa et al., 2005, 2007; Perez et al., 2001). Moreover, such neuronal plasticity associated with spatial learning may not be restricted to the CA1 region and may involve structural changes as well. Indeed, recently it has been discovered that spatial learning triggers an increase in the numbers of filopodial synapses from hippocampal mossy fibers onto fast-spiking interneurons (Ruediger et al., 2011).

In our analysis, we identified factors that promote these connection changes. We have found that the pairing of the pre- and postsynaptic action potentials measured during learning was important, and that the change in connection strength was stronger when the presynaptic pyramidal cell fired at times when the postsynaptic interneuron was strongly active. This is in agreement with the finding that the pairing of presynaptic action potentials with the depolarization of postsynaptic interneurons initiate synaptic plasticity for certain cell types (Lamsa et al., 2005, 2007). Here, we also show that spike pairing is more effective when it takes place near goal locations. At these locations several factors could have promoted plastic changes including reward-related release of dopamine and waking SWRs firing synchronization of pyramidal cells.

In summary, this work demonstrates the spatial learning-related reorganization of connections from pyramidal cells to interneurons in the CA1 region. Such reconfiguration of the hippocampal interneuron circuit may support spatial learning in a wide variety of ways including modulation of pyramidal cell spike timing and local neuronal plasticity. Moreover, it can help to maintain the integrity of hippocampal maps while still labile, or compensate for the reorganization of pyramidal excitatory circuits and alleviate the problem of interference between maps. Finally, our findings show that learning and memory processes engage wide ranging modification of hippocampal circuits including not only pyramidal circuits but that of interneurons onto which pyramidal assemblies synapse.

## Experimental Procedures

### Subjects and Electrode Implantation

All procedures were carried out in accordance with the Animals (Scientific Procedures) Act, 1986 (UK), and associated procedures under an approved project license. A total of ten adult male Long-Evans rats (Harlan, UK) were implanted with 16 independently movable wire tetrodes that were positioned above the right dorsal hippocampus (see Supplemental Information). Rats were housed individually in standard rodent cages (56 × 40 × 26 cm) in a temperature and humidity controlled animal room. They were maintained on a 12 hr light/dark cycle and all testing performed during the light phase. Food and water were available ad libitum prior to the recording procedures and body weight at the time of surgery was 350–400 g.

### Behavior

Animals were trained to perform a spatial learning task on a cheeseboard maze as previously described (Dupret et al., 2010). In this task, animals had to learn three new goal locations where food reward were hidden every day. Each daily experiment consisted of a sequence of five recording sessions during which neuronal assembly activity was continuously monitored: a prelearning probe test (“preprobe”), a prelearning immobility/sleep rest session (“presleep”), a learning session, a postlearning immobility/sleep rest session (“postsleep”), and a postlearning probe test (“postprobe”) (see Supplemental Information). The two probe tests (∼25 min) were never rewarded. After both the preprobe and the learning sessions, rats were allowed to settle down within the start box for the rest sessions (∼25 min). During the learning session, rats were given successive trials (∼40 trials) to locate a new set of three hidden rewards placed in randomly selected food wells every day. As these baited locations changed from day to day but stayed fixed within a given day, this “matching-to-multiple-places” procedure required frequent updating of memory for goal locations in an otherwise unchanging environment.

### Unit Isolation

Unit isolation and clustering procedures have been described previously (Csicsvari et al., 1998, 1999; O’Neill et al., 2008). Briefly, the continuously recorded wide-band signals were digitally high-pass filtered (0.8–5 kHz). The power (root mean square) of the filtered signal was computed in a sliding window (0.2 ms) for spike detection. The standard deviation (SD) was calculated to estimate the variance of the baseline noise and to establish a detection threshold. Action potentials with a power of more than five times the SD from the baseline mean were selected. The spike features were then extracted by using principal components analyses. The detected action potentials were then segregated into putative multiple single units by using automatic clustering software (Harris et al., 2001; http://klustakwik.sourceforge.net/). Finally, the generated clusters were manually refined by a graphical cluster cutting program (Csicsvari et al., 1998). Only units with clear refractory periods (<2 ms) in their autocorrelation and well-defined cluster boundaries (Harris et al., 2001) were used for further analysis. Pyramidal cells and interneurons were discriminated by their autocorrelations, firing rates and wave forms, as previously described (Csicsvari et al., 1999). Because our goal was to analyze changes in the hippocampal firing patterns over different time points, we needed to ensure that our sample of cells was taken from clusters with stable firing. We therefore clustered together periods of waking spatial behavior and sleep sessions. Stability of the recorded cells over time was verified by plotting spike features over time and by plotting two-dimensional unit cluster plots in different sessions in addition to the stability of spike waveforms. In addition, an isolation distance based on Mahabalonis distance was calculated to ensure that the selected spike clusters did not overlap during the course of the recordings (Harris et al., 2001). In total, 2,319 pyramidal cells and 302 interneurons from the CA1 region of the hippocampus recorded in the “allocentric learning” version of the task, and 153 CA1 interneurons recorded in the “cued learning” version, were included in the analysis.

### Pyramidal Cell Assembly Expression

Hippocampal place rate maps were calculated during exploratory epochs (speed > 5cm/s) as described before (Dupret et al., 2010; O’Neill et al., 2008). Place cells were then screened for their spatial tuning using a coherence value of at least 0.6 and a sparsity value of no more than 0.3. Coherence reflects the similarity of the firing rate in adjacent spatial bins and is the z transform of the correlation between the rate in a bin and the average rate of its eight nearest neighbors (Muller and Kubie, 1989). Sparsity corresponds with the proportion of the environment in which a cell fires, corrected for dwell time (Skaggs et al., 1996), and is defined as (ΣPiRi)^2^/ΣPiRi^2^, where Pi is the probability of the rat occupying bin i, Ri is the firing rate in bin i. The expression of pyramidal cell assembly patterns was estimated using a population vector-based analysis (Dupret et al., 2010; Leutgeb et al., 2005) in a subsecond time scale. The rate maps of CA1 pyramidal cells were stacked into three-dimensional matrices (the two spatial dimensions on the x and y axis, the cell identity on the z axis; see Figure 2A) for the preprobe and the postprobe sessions. In these sessions each x-y bin was thus represented by a population vector composed by the firing rate of each pyramidal cell at that location. The number of pyramidal cells used was at least 14 and up to 71, with a median at 40 cells. The detection of theta-oscillatory waves was performed as previously described (Csicsvari et al., 1999; O’Neill et al., 2006) by filtering the local field potential (5–28 Hz) and detecting the negative peaks of individual waves. Theta cycles that were detected globally using all electrodes located in CA1 and identified in each learning trial, were used as time windows to calculate the instantaneous firing rate of the pyramidal neurons and establish a population vector. Each of these vectors during learning was correlated with the corresponding x-y vector representing the same location during the probe session before and after learning. A Fisher z-test was then used to test the null hypothesis that the correlation between the assembly patterns in learning and those expressed in the preprobe was the same as the correlation between the assembly patterns during learning and those expressed during the postprobe (Fisher, 1921; Zar, 1999). The z values obtained from this procedure that compares pairs of population vector correlations in each theta cycle allow assessing the ongoing expression of hippocampal maps: positive values indicate times at which the pyramidal activity patterns preferentially expressed the new cell assemblies developed during learning, while negative values suggest the expression of the old pyramidal assemblies. Standard errors were used when population means were compared.

### Firing Associations to Pyramidal Assemblies

To measure the firing association of interneurons and pyramidal cells to the expression of pyramidal assemblies, the instantaneous firing rate (IFR, in Hz) of each neuron was calculated during learning for each theta cycles used as time window for the analysis. Then the association of each cell was measured by calculating the correlation coefficient (Pearson-moment product) between the IFR and the z value of the assembly expression measured in the same window. However, we ensured that each pyramidal cell’s own activity did not influence the assessment of its assembly membership. To do so, we left out that cell from the population vector used for determining which cell assembly was expressed. Using the last 10 learning trials cells that exhibited significant correlations (p < 0.05) were divided by whether they exhibited positive or negative correlation coefficients. The firing associations to the new assemblies were confirmed using a logistic regression between the IFR and the time windows in which the newly-established cell assemblies were present (critical value: α > 1.960) (Zar, 1999).

### Pyramidal Cell-Interneuron Coupling

Isolation of monosynaptically-connected pyramidal cell-interneuron pairs were performed as described previously by identifying cross-correlograms between pyramidal cells and interneurons that exhibited a large, sharp peak in the 0.5–2.5 ms bins (after the discharge of the reference pyramidal cells) (Csicsvari et al., 1998). Because the number of action potentials used for the construction of these cross-correlograms varied from cell to cell, the histograms were normalized by dividing each bin by the number of reference pyramidal spike events (Csicsvari et al., 1998). The connection strength was thus accessed by measuring the spike transmission probability at the monosynaptic peak indicating the probability that the pyramidal cell would discharge its postsynaptic interneuron partner. However, the chance probability of the two cells firing together was subtracted in order to account for firing rate change-related fluctuations in the correlation strength. The chance firing probability was estimated by averaging the 30–50 ms bins in both sides of the histogram. The significance level for the monosynaptic peak was set at three standard deviations from the baseline (p < 0.000001) (Abeles, 1982; Csicsvari et al., 1998). In a further analysis, the correlation coefficient of pyramidal cell-interneuron spike coincidence was calculated instead of spike transmission probability on the cross-correlation histograms where pyramidal cell spikes were still used as reference (see Figures S6C–S6H). For this the spike train covariance function was divided by the square root of standard deviation of the firing rates of both cells. Correlation coefficients of spike coincidence hence provide an additional measure independent of the firing rate of both cells to assess pyramidal cell-interneuron coupling strength.

### Definition of Behavioral States and Detection of Oscillatory Waves

Recordings sessions were segregated off-line onto periods of exploratory activity and rest (immobility/sleep) as previously described (Csicsvari et al., 1998, 1999; O’Neill et al., 2006). For each session, the theta/delta ratio was plotted against speed so that the behavioral state could be manually identified. The theta/delta power ratio was measured in 1,600 ms segments (800 ms steps between measurement windows), using Thomson’s multitaper method (Mitra and Pesaran, 1999; Thomson, 1982). Exploratory epochs included periods of locomotion and/or the presence of theta oscillations (as seen in the theta/delta ratio), with no more than 2.4 s (i.e., two consecutive windows) of transient immobility. Rest epochs were selected when both the speed and theta-delta ratio dropped below a pre-set threshold (speed: <5cm/s, theta/delta ratio: <2) for at least 2.4 s. During periods of active waking behavior, theta-oscillatory waves detection was performed as previously described (Csicsvari et al., 1999; O’Neill et al., 2006) using the negative peaks of individual theta waves from the filtered trace of the local field potential (5–28 Hz). The band used for the detection was wider than the theta band in order to precisely detect the negative peaks of the theta waves, which otherwise would have smoothed out in using a narrow theta band. For gamma-oscillatory wave detection, local field potentials were band-pass filtered (30–80 Hz) and the power (root mean square) of the filtered signal was calculated for each electrode as previously described (Csicsvari et al., 2003; Senior et al., 2008). For the detection of SWRs, local field potentials were band-pass filtered (150–250 Hz), and a reference signal (from a channel that did not contain ripple oscillations) was subtracted to eliminate common-mode noise (such as muscle artifacts). The power (root mean square) of the filtered signal was calculated for each electrode and summed across electrodes designated as being in the CA1 pyramidal cell layer. The threshold for SWR detection was set to 7 SD above the background mean. The SWRs detection threshold was always set in the first sleep session, and the same threshold was used for all other sessions. The SWR firing rate histograms of pInt and nInt interneurons were calculated during the sleep session before learning using 20 ms bin in reference to the SWR peak (i.e., peak of ripple-band power) as previously described (Dupret et al., 2010; O’Neill et al., 2006).

## Supplemental Information

Document S1. Figures S1–S8 and Supplemental Experimental Procedures
