# Supplementary material for: Dynamic Reconfiguration of Hippocampal Interneuron Circuits during Spatial Learning
Source: Neuron. 2013 Apr 10;78(1):166–80. doi: 10.1016/j.neuron.2013.01.033 (PMC3627224; doi:10.1016/j.neuron.2013.01.033)
Supplement: Document S1. Figures S1–S8 and Supplemental Experimental Procedures [file mmc1.pdf]

Neuron, Volume *78*

Supplemental Information

Dynamic Reconfiguration of Hippocampal Interneuron Circuits during Spatial Learning

David Dupret, Joseph O'Neill, and Jozsef Csicsvari

Inventory of Supplemental Information

Contains 8 Supplemental Figures, Experimental Procedures and References.

- Figure S1, linked to Figure 1,3: Experimental layout of the cheeseboard maze task and rate changes of interneurons.

- Figure S2, linked to Figures 1,3,4: Cued learning version of the cheeseboard maze task. This figure shows that changes of both interneuron rate and pyramidal cell-interneuron coupling strength were not observed when the animals performed the task without the allocentric learning context, as tested in the cued condition where reward locations were indicated by intra-maze cues.

- Figure S3, linked to Figure 2: Dynamic expression of pyramidal cell assemblies during learning. This figure illustrates that new pyramidal cell assemblies expressed in theta or gamma cycles alternated with previously established ones, and eventually overtook them at the end of the learning.

- Figure S4, linked to Figure 3: Interneuron firing associations to pyramidal cell assemblies at different locations and different speeds values. This figure shows that the firing associations of interneurons to pyramidal assemblies were not influenced by place; indeed, these associations measured in different locations (i.e. path segments between goal locations) were similar, indicating that they were independent of space. It also shows that these firing associations were not influenced by animal's speed.

- Figure S5, linked to Figures 3-5: Theta, gamma and sharp wave-ripple oscillations response of pInt and nInt interneurons. This figure shows that pInt and nInt interneuron groups exhibited certain physiological differences beyond their association to pyramidal assemblies.

- Figure S6, linked to Figure 4: Change in pyramidal cell-interneuron connection strength. This figure gives examples of monosynaptically-connected pyramidal cell-interneuron pairs as identified by the presence of a sharp peak at short-latency in cross-correlation histograms. Its also shows that analysis of pyramidal cell-interneuron cross-correlograms using spike correlation coefficients provide an additional measure independent of the firing rate of both cells to assess pyramidal cell-interneuron coupling strength.

- Figure S7, linked to Figure 4-6: This figure shows that the changes in spike transmission probability were not related to the preferred theta phase of pyramidal cells and interneurons pairs.

- Figure S8, linked to Figure 4-6: Theta phase of pyramidal cell-interneuron monosynaptic events. This figure shows that changes in spike transmission probability were not related to changes in theta phase of spike transmission events.

1. Supplemental Figures

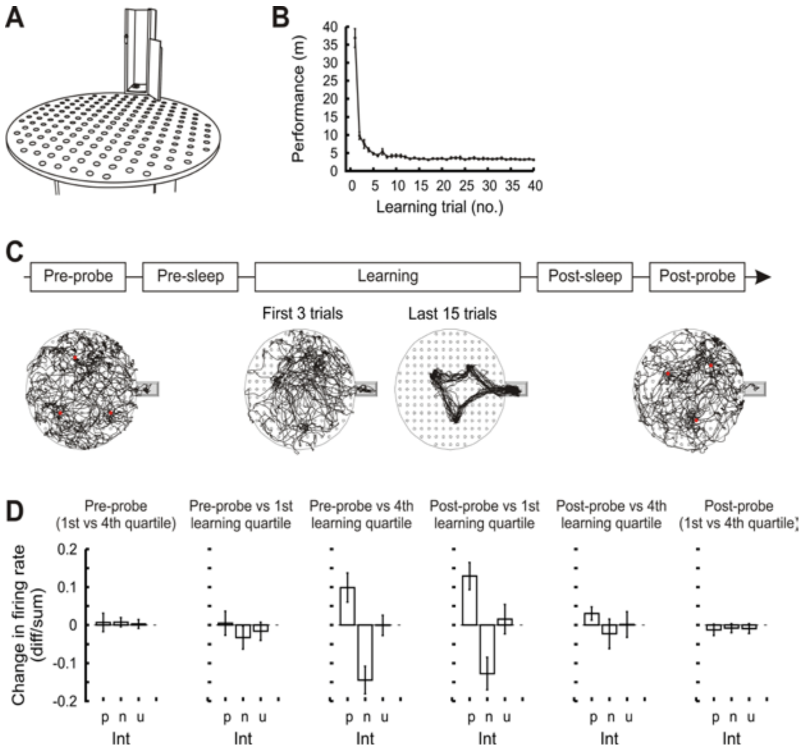

Figure S1. Experimental layout of the cheeseboard maze task and rate changes of interneurons.

(A) Schematic illustration of the behavioral apparatus including the cheeseboard maze and the connected start box.

(B) Learning performance estimated from the distance travelled to retrieve all rewards during each trial (mean±s.e.m, $P<0.00001$, ANOVA).

(C) (upper) Schematic of the experimental design indicating pre- and post-probe assessment of the animals memory for goal locations, pre- and post-learning sleep sessions, and the learning period and (lower) representative examples of an animal's behavioral trajectory during probe and learning sessions within a given recording day; for clarity only the first 10 min of each probe session are depicted (red dots: learned goal locations).

(D) Change in firing rate of different interneuron groups (mean±s.e.m) measured as the rate difference divided by the sum within the probe sessions (1st versus 4th quartile) or between the probe sessions and learning (1st or 4th quartile). The pInt and the nInt interneurons exhibited significant rate changes for pre-probe versus 4th learning quartile and post-probe versus 1st learning quartile (all $Ps<0.005$; in the other cases: all $Ps>0.124$).

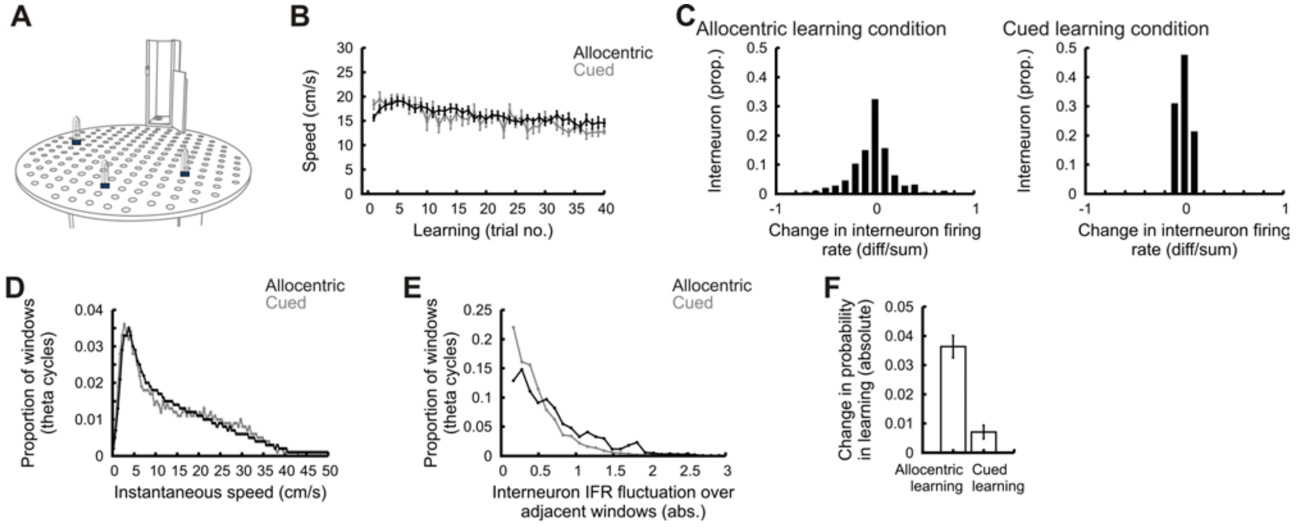

Figure S2. Cued learning version of the cheeseboard maze task.

(A) Schematic illustration of the behavioral apparatus including the cheeseboard maze with the intra-maze cues and the connected start box.

(B) Average speed of rats trained to locate hidden food rewards in the absence ("allocentric" condition) or presence ("cued" condition) of intra-maze cues marking the goal locations (P=0.106, ANOVA).

(C) Distribution of the normalized change in interneuron firing rate during both "allocentric" and "cued" learning conditions. The rate change was measured as the signed difference between the rates in the first 10 min and the last 10 min of learning, divided by their sum.

(D) Distribution of the instantaneous speed measured in time windows (theta cycles) during both "allocentric" (black) and "cued" (grey) learning conditions (D=0.100, P=0.676, Kolmogorov-Smirnov test).

(E) Distribution of the interneuron's instantaneous rate fluctuations ("IFR") in adjacent time windows (theta cycles) during both "allocentric" (black) and "cued" (grey) learning conditions (D=0.393, P=0.0081, Kolmogorov-Smirnov test). Note that the instantaneous rate fluctuations of interneurons over adjacent theta cycles were smaller during the cued task than in the allocentric one, while in both conditions the task was performed with similar movement paths and at similar instantaneous speeds. Therefore, rate fluctuations of interneurons associated with allocentric learning were bigger than those that could be expected due to changes in locomotor or spatial behavior or by natural intrinsic variability.

(F) Absolute change in spike transmission probability during learning (1st versus 4th quartile). The spike transmission peak probabilities were calculated at the monosynaptic delay period (0.5–2.5ms bins) from the pyramidal cell-interneuron cross-correlograms using all cell pairs exhibiting a significant peak to compare the two learning conditions (P=0.037, t-test).

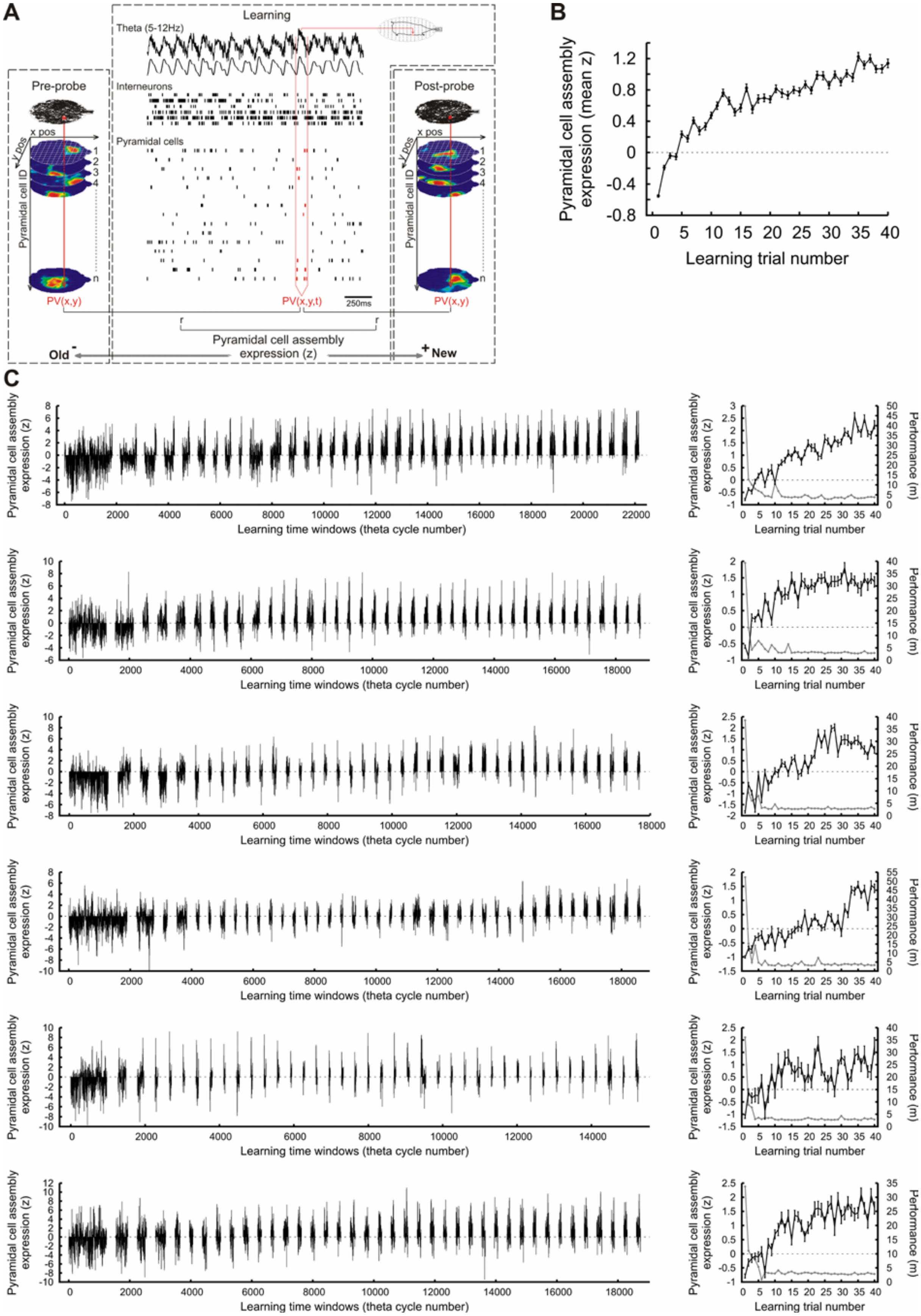
A
Learning
Theta (5-12Hz)
Interneurons
Pyramidal cells
Pre-probe
Post-probe
x pos
y pos
Pyramidal cell ID
PV(x,y)
PV(x,y,t)
250ms
r
Pyramidal cell assembly
expression (z)
Old
New
B
Pyramidal cell assembly
expression (mean z)
Learning trial number
C
Pyramidal cell assembly
expression (z)
Learning time windows (theta cycle number)
Learning trial number
Performance (m)

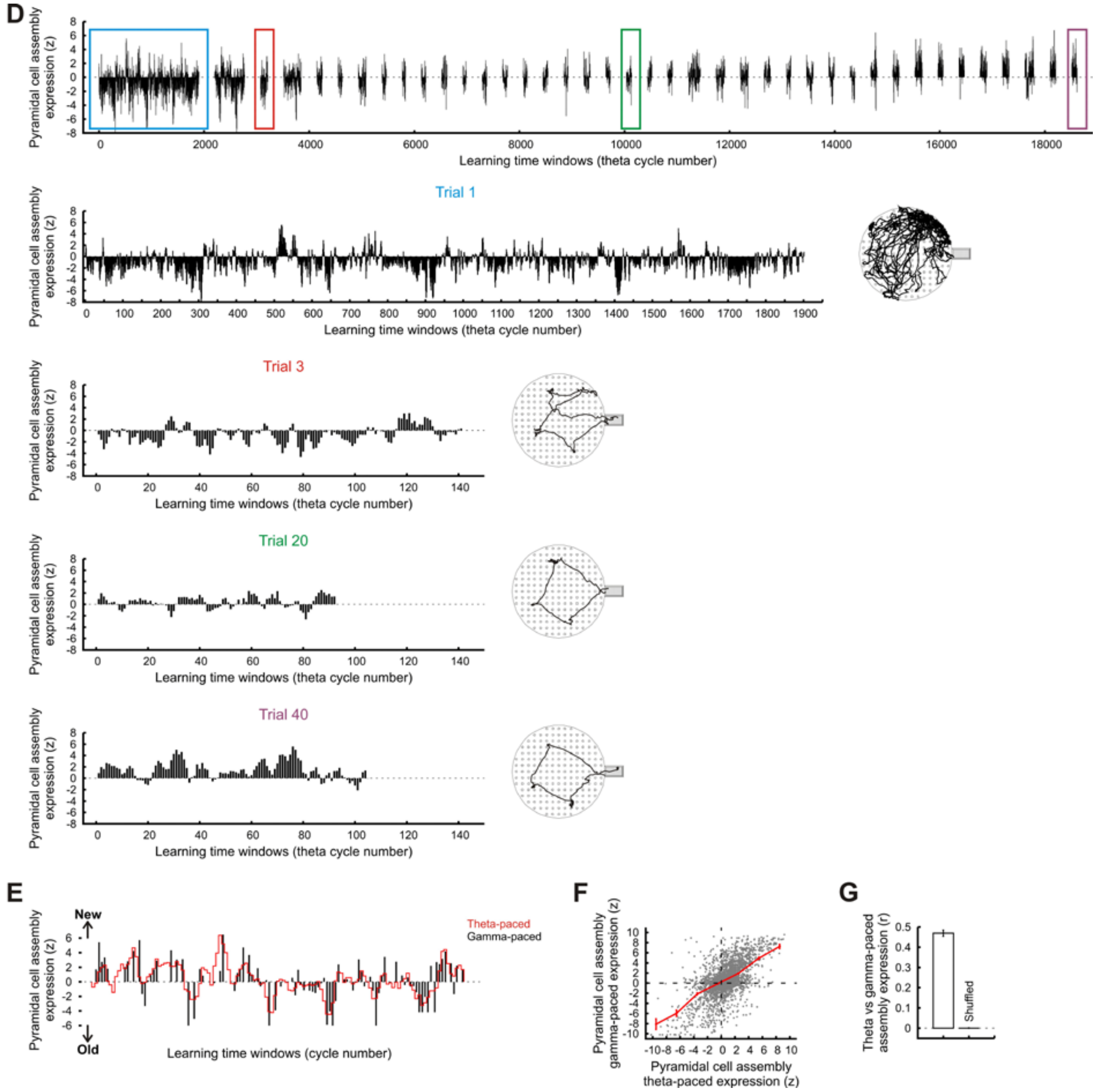

Figure S3. Dynamic expression of pyramidal cell assemblies during learning

(A) Schematic illustration of the population vector-based analysis used to establish the pyramidal cell assembly expression in theta cycles during learning. The rate maps of CA1 pyramidal cells were stacked into three-dimensional matrices for each probe session (the two spatial dimensions on the x and y axis, cell identity on the z axis) so that each x-y bin was represented by a population vector composed of the firing rate of each cell at that location. Next, theta cycles in each learning trial were identified and used as time windows to calculate the instantaneous spike counts of the pyramidal cells and establish a population vector. Each of these theta vectors was correlated with the corresponding x-y vector representing the same location from the probe session before and after learning (Pearson correlation); then a Fisher z-test was performed to estimate whether the ongoing population vector during learning was more similar to the one expressed before or the one expressed after (Fisher,

1921;Zar, 1999). Positive z values indicate times at which the pyramidal activity patterns preferentially expressed the new cell assemblies developed during learning while negative values indicate times when the old pyramidal assemblies were expressed.

(B) Mean pyramidal cell assembly expression showing the shift from population activity from one more consistent with old (pre-probe) cell assembly patterns, as denoted by the negative z, to positive z values reflecting assembly activity increasingly aligned with new (post-probe) assembly patterns as the number of learning trials increases (mean z±s.e.m).

(C) Individual examples of pyramidal cell assembly expression measured in theta cycles during learning (left panels) and averaged per trial (black curve on the right panels, "mean±s.e.m") together with the behavioral performance per trial (grey curve on the right panels) during learning. Behavioral performance was estimated by the distance travelled (in meters) to find the three goal locations on each trial. Each row represents one example learning session. Note that both the new (positive z) and the old (negative z) assemblies are expressed on each trial but later trials are dominated by the new assembly patterns.

(D) Individual example illustrating the presence of both new and old assemblies in non-overlapping theta cycles for one learning session. The first row depicts the pyramidal cell assembly expression across the entire learning session; below this are learning trials 1, 3, 20 and 40 given at a higher magnification together with the behavioral trajectory of the animal during the given trial (right panels). Note that within each trial the old and the new pyramidal assemblies dynamically switched from one to another with the new assemblies dominating late trials. Note also that the new assembly expression gets stronger in the later trials even when the animal's paths remain similar (trial 20 versus trial 40).

(E-G) Theta- and gamma-paced flickering of pyramidal cell assemblies. The expanded trace shows that flickering between the new and the old pyramidal cell assemblies in non-overlapping theta cycles (red line) extends to the gamma oscillations timescale (black impulses) during learning (**E**). The scatter plot shows that gamma-paced cell assembly expression correlated with theta-paced assembly expression during a learning session (**F**, $r=0.639$, $P<0.00001$). Such correlations of gamma-paced and theta-paced assembly expressions were not observed when the pyramidal cell assembly expression values obtained in gamma and theta cycles were shuffled relative to each other (**G**, mean r±s.e.m, $P<0.00001$).

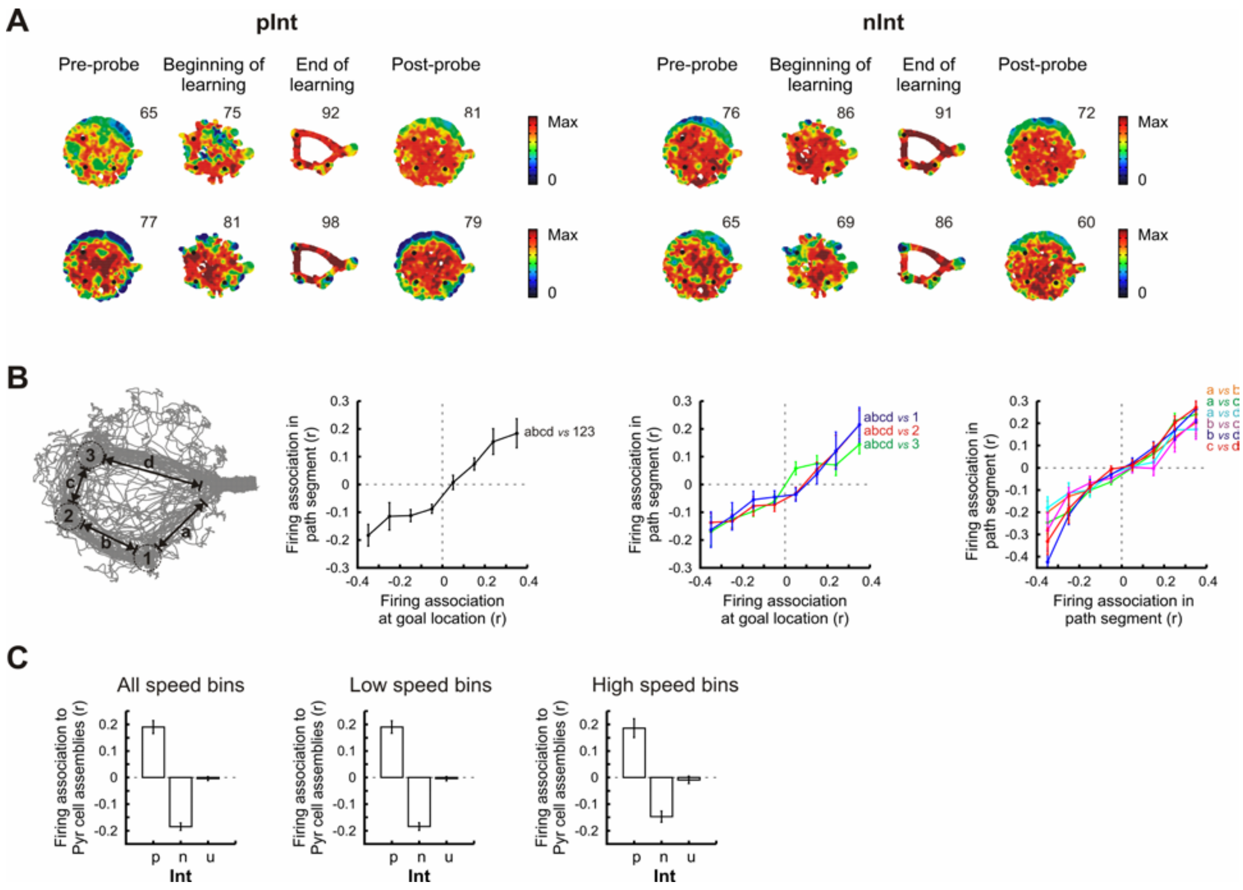

Figure S4. Interneuron firing associations to pyramidal cell assemblies at different locations and different speeds values

(A) Examples of the place rate maps of 2 pInt (left) and 2 nInt (right) interneurons. Each row shows color-coded maps (with peak rate values indicated) for both pre- and post-probe sessions and averaged place rate maps from the beginning (first 3 trials) and the end (final 10 trials) of a learning session. Black dots indicate goal locations.

(B) The firing associations of p/nInt interneurons to pyramidal cell assemblies measured at different locations of the cheeseboard maze during learning were similar and were found to be significantly correlated (all Rs>0.385, all Ps<0.013, Pearson correlations) indicating that the firing association of interneurons to pyramidal assemblies appears to be independent of location or position of the animal. The firing associations were calculated for either all or individual goal locations (goal location “1”, “2” and “3”) and for all or individual path segments between goal locations (path segment “a”, “b”, “c” and “d”). Note that **(A)** and **(B)** suggest that the firing associations of interneurons to pyramidal assemblies were also not influenced by place: firing associations measured in different locations (i.e. path segments between goal locations) were similar, indicating that they were independent of space.

(C) Interneuron firing associations to pyramidal cell assemblies were consistent across different speeds values (p/nInt: all $Ps<0.0013$; uInt: all $Ps>0.60$) indicating that these associations also occurred independently of the speed of the animal. The threshold for low versus high speed bins was set at 8cm/sec.

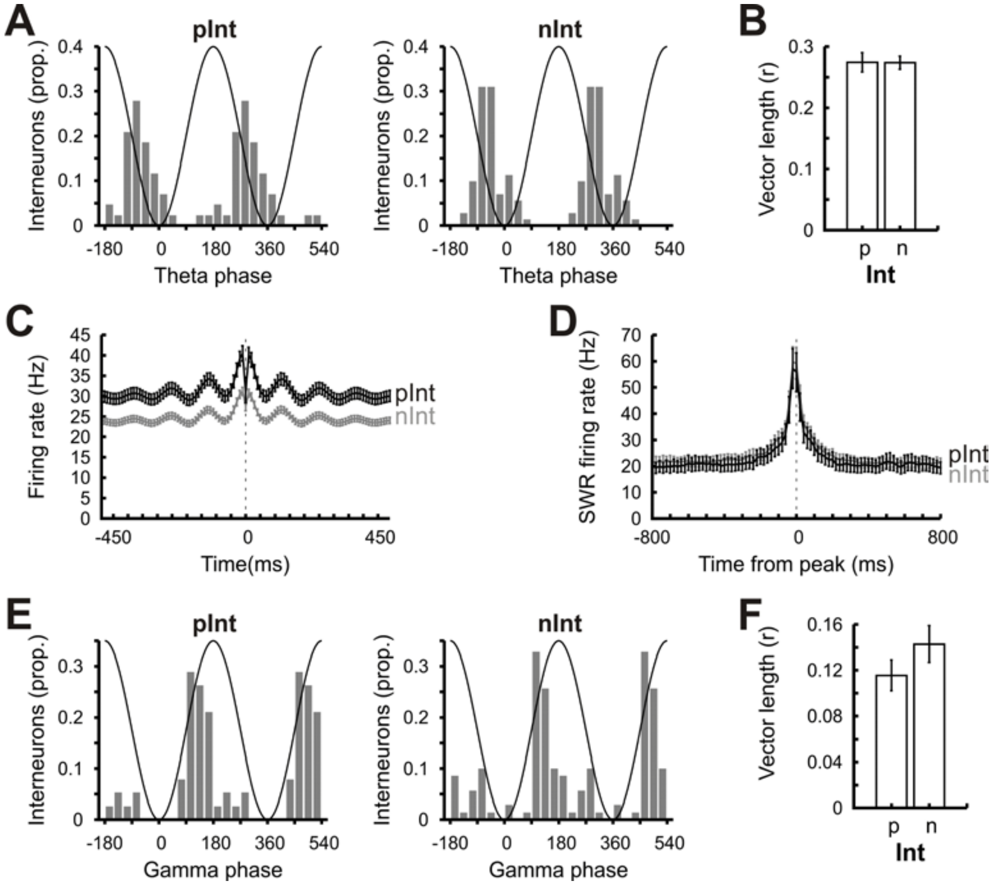

Figure S5. Theta, gamma, and sharp wave-ripple oscillations response of pInt and nInt interneurons.

(A-B) Distribution of the preferred theta (5–12 Hz) phase of interneuron spikes (**A**, P<0.024, Watson-Williams test) and their mean depth of modulation by theta oscillations (**B**, Rayleigh vector length, P=0.965, t-test).

(C) Average autocorrelation histograms for pInt and nInt interneurons calculated during learning (mean ±s.e.m; 10ms bin). Note that both types of interneurons exhibit strong theta-modulation.

(D) Sharp wave-ripple (150–250 Hz) firing rate histograms (mean±s.e.m) of pInt and nInt interneurons. Firing rates were measured in 20ms bins in reference to SWR peaks. SWR peak responses of pInt and nInt interneurons were similar (55.44±6.66 Hz versus 57.80±8.31 Hz, P=0.824, t-test).

(E-F) Distribution of the preferred gamma (30–80 Hz) phase of interneuron spikes (**E**, P=0.730, Watson-Williams test) and their mean depth of modulation by gamma oscillations (**F**, Rayleigh vector length, P=0.095, t-test).

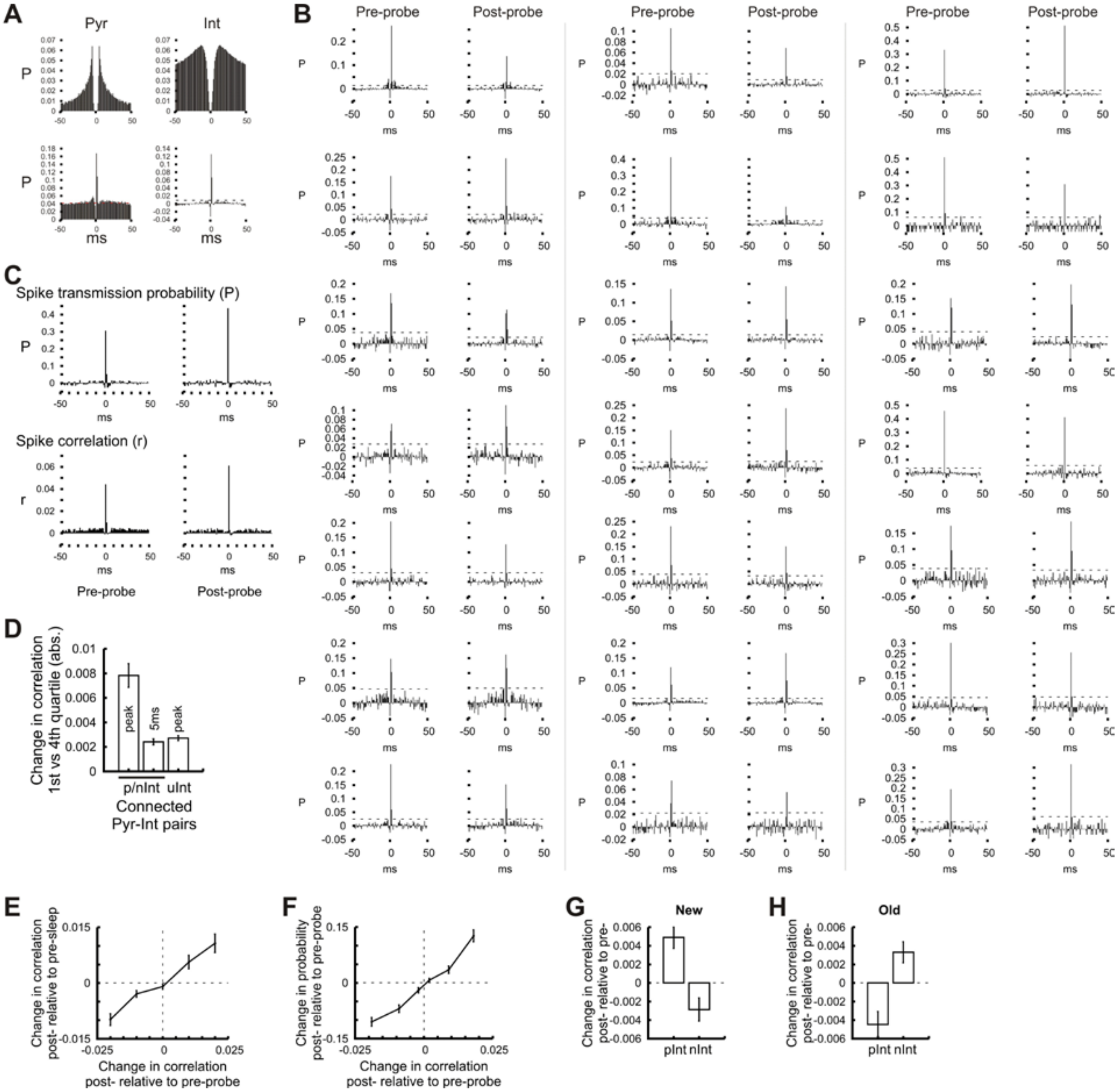

Figure S6. Change in pyramidal cell-interneuron connection strength

(A) Auto-correlograms for a pyramidal cell (top left histogram, “Pyr”) and its connected interneuron (top right histogram, “Int”) and their cross-correlograms (bottom histograms). In these cross-correlograms the pyramidal cell action potentials were used as reference. The joint probability that the two cells fire together by chance was calculated in the first and last 20 ms bins in the original cross-correlogram (bottom left histogram) and subtracted in order to remove firing rate change-related fluctuations in the correlation strength (bottom right histogram). Note the presence of a large, sharp peak at short-latency (<3 ms) as the signature of an excitatory monosynaptic connection. Note also the

absence of theta modulation in the cross-correlograms. Dashed lines represent 3 SD from the mean firing probability.

(B) Examples of pyramidal cell-interneuron cross-correlograms for pre- and post-probe sessions. Note that the spike transmission probability can decrease or increase from the pre-probe to the post-probe.

(C) Examples of cross-correlograms using spike correlation coefficients (bottom row) for one pyramidal cell-interneuron pair during the pre-probe (left) and the post-probe (right) sessions. The cross-correlograms of the same cell pair established using spike transmission probability are depicted for comparison (top row). Cross-correlograms using the correlation coefficient of pyramidal cell-interneuron spike coincidence were calculated with the pyramidal cell action potentials used as reference. For this the spike train covariance function was divided by the square root of standard deviation of the firing rates of both cells. Correlation coefficients of spike coincidence therefore provided an additional measure of pyramidal cell-interneuron coupling strength that was independent of the firing rate of both cells.

(D) Absolute change in spike correlation (mean±s.e.m) during learning (1st versus 4th quartile) for cell pairs of pyramidal cells connected to interneurons of the different groups (p/nInt and uInt). The correlation coefficients were calculated at the peak and 5ms bins. This analysis confirms that spatial learning is associated with changes in pyramidal cell-p/nInt interneuron coupling strength as shown by an increased absolute change in spike correlation during the monosynaptic period only ($P<0.0001$, ANOVA).

(E) Change in pyramidal cell-interneuron spike correlation across the sleep sessions as a function of the change in correlation across probe sessions (mean±s.e.m; $r=0.181$, $P<0.03$).

(F) Change in pyramidal cell-interneuron spike transmission probability as a function of the change in spike correlation (mean±s.e.m; $r=0.695$, $P<0.0001$). Changes were calculated across the probe sessions.

(G,H) Change in pyramidal-interneuron spike correlation (mean±s.e.m) from the pre-probe to the post-probe according to the pyramidal cell assembly membership. This analysis confirms that pyramidal cells that were members of a new assembly strengthened their connections to pInt interneurons and weakened their connections to nInt interneurons (G, all $Ps<0.028$); the opposite changes were observed with pyramidal cells linked to the old assemblies (H, all $Ps<0.030$).

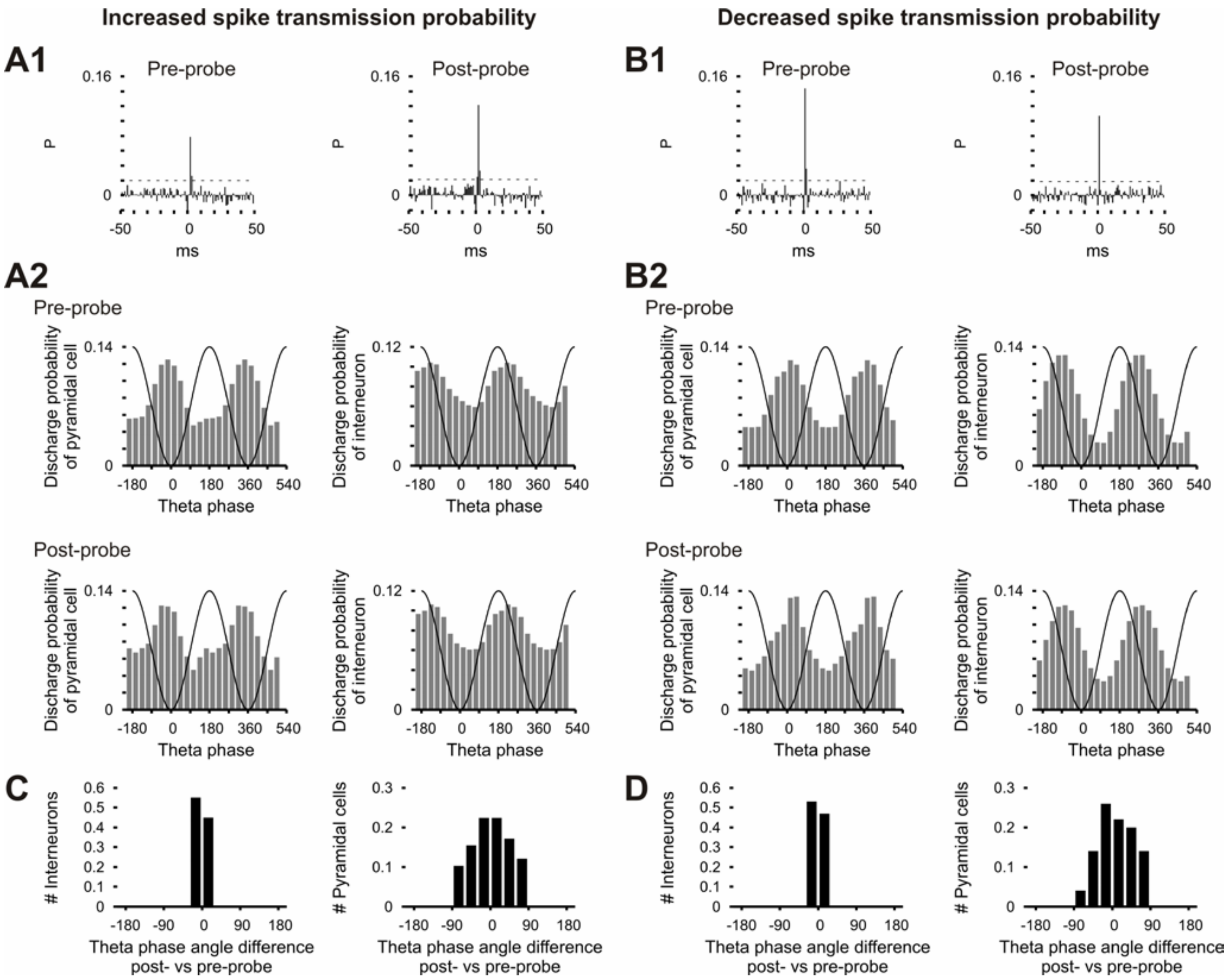

Figure S7. The changes in spike transmission probability are not related to the preferred theta phase of pyramidal cells and interneurons pairs

(A) Example of an increased spike transmission probability at a pyramidal cell-interneuron pair. Note the increase in probability at the monosynaptic delay (0.5–2.5 bins) when comparing the cross-correlograms from the pre-probe and the post-probe sessions (**A1**). Note also the similar theta phase distributions of firing probabilities during the pre-probe (**A2**, top) and the post-probe (**A2**, bottom) sessions for both the pyramidal cell (**A2**, left) and the interneuron (**A2**, right).

(B) Example of a decreased spike transmission probability at a pyramidal cell-interneuron pair. Note the decrease in probability at the monosynaptic delay when comparing the cross-correlograms from the pre-probe and the post-probe sessions (**B1**). Note also the similar theta phase distributions of firing probabilities during the pre-probe (**B2**, top) and the post-probe (**B2**, bottom) sessions for both the pyramidal cell (**B2**, left) and the interneuron (**B2**, right).

(C,D) Distributions of the change in the preferred theta phase (angle difference, post-probe relative to pre-probe) for interneurons (left panel in **C** and **D**) and pyramidal cells (right panel in **C** and **D**) that are members of connected pairs which spike transmission has increased (**C**) or decreased (**D**) after learning. Note the similar distributions of theta phase angle difference for both the pyramidal cells and the interneurons (increased spike transmission probability versus decreased spike transmission probability, all Ps>0.991, Kolmogorov-Smirnov test). Note also that the changes in pyramidal cell-interneuron spike transmission probability were not correlated with the change in theta phase angle difference of either the interneurons or the pyramidal cells (all Ps>0.19).

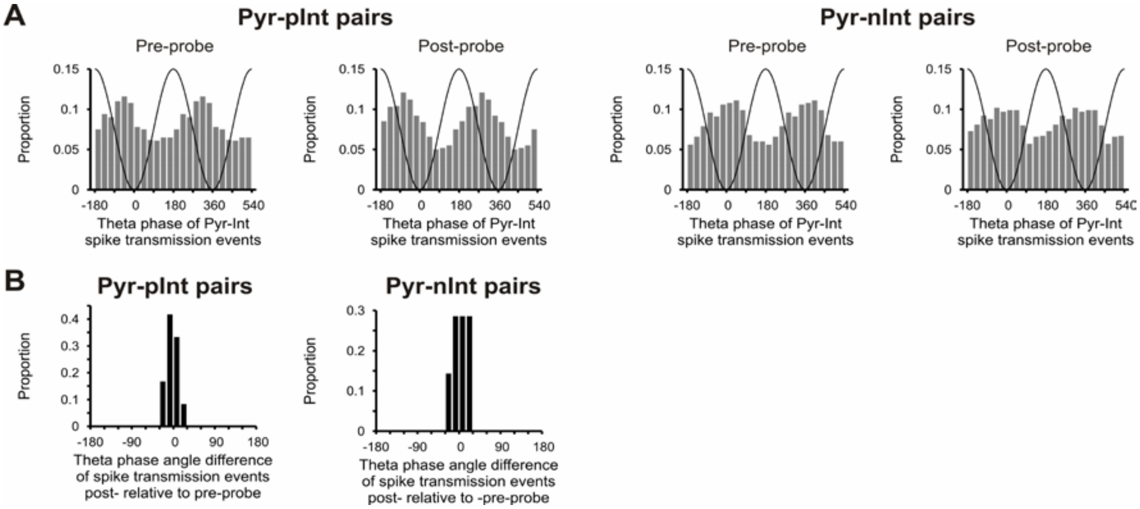

Figure S8. Theta phase of pyramidal cell-interneuron monosynaptic events

(A) Distribution of the preferred theta phase of pyramidal cell-interneuron monosynaptic events during the pre-probe and the post-probe sessions for pyramidal cells connected to a pInt (left) or nInt (right) interneuron. Note that the preferred theta phase at which the pyramidal cell-interneuron spike transmission events occurred did not change from the pre-probe session prior to learning to the post-probe session following learning.

(B) Distribution of change in the preferred theta phase of pyramidal cell-interneuron monosynaptic events (theta angle difference) from pre-probe to post-probe. The change in pyramidal cell-interneuron spike transmission probability was not correlated with the change in theta phase of these transmission events (r=0.08, P=0.417, linear-circular correlation).

2. Supplemental Experimental Procedures

Apparatus. The behavioral apparatus used for these experiments (see **Figure S1**) was a circular arena referred to as the cheeseboard maze (Dupret et al., 2010;Gilbert et al., 1998;Kesner et al., 1991). The board (120 cm in diameter, 2 cm in thickness) was made of plastic, painted black, and stood 55 cm above the floor in the recording room. A total of 177 food wells (2.5 cm in diameter, 1.5 cm in depth) were drilled into the surface of the maze in evenly spaced parallel rows and columns (8 cm between the centers of each well). A wood-made black start-box (27 cm long, 19 cm wide and 59 cm high) was placed along the edge of the board perpendicular to the rows of food wells. The top of the box was open to allow the tracking of the animal inside; the floor was covered by brown cardboard and the front was equipped with a door (35 cm high).

Surgery. The surgical and recording procedures, electrode preparation, implantation, and spike sorting methods have been described before (O'Neill et al., 2006;O'Neill et al., 2008). In short, rats were implanted with 16 independently movable wire-tetrodes under deep anesthesia using isoflurane (0.5-2 %), oxygen (2 l/min) and an initial dose of buprenorphine (0.1 mg/kg). Wire tetrodes constructed from 12 µm diameter tungsten wires (H-Formvar insulation with Butyral bond coat, California Fine Wire, Grover Beach CA) were attached to the microdrives, enabling their independent movement. The tetrode tips were gold-plated to reduce electrode impedances to 250-500kΩ. During surgery, a craniotomy was performed above the right dorsal portion of the hippocampus, centered at AP=-3.8; ML=2.5, the dura mater was removed, and the electrode bundles implanted into the superficial layers of the neocortex, after which both the exposed cortex and the electrode shanks were sealed with paraffin wax. Two micro-screws (M1.4), placed above the cerebellum, served as ground and reference electrodes. Following a one-week postoperative recovery period, rats were reduced to and maintained at 85% of their age-matched preoperative weight. Water was available *ad libitum*. During this period, the tetrodes were lowered into the CA1 region of the dorsal hippocampus (close to the *stratum pyramidale*) over a further period of approximately 7 days.

Data acquisition. Wide-band (0.1Hz–5 kHz) recordings of local field potentials and multiple-unit activity were amplified 1,000-fold using a 64-channel amplifier (Sensorium) and continuously digitized at 20 kHz using a 64-channel analogue-to-digital converter computer card (United Electronics Industries). Two 32-channel unity-gain preamplifier headstages (Axona Ltd, www.axona.com) were

used to reduce cable movement artefacts. An array of three LED clusters mounted on the housing containing the electrode arrays were used to track the location of the animal (25 frames per s) via an overhead video camera (Sony). The animal tracking was synchronized with the electrophysiological recording. The animal's location was constantly monitored throughout the daily experiment. The data were analyzed off-line using custom-made software.

Experimental design. Rats were trained to locate the hidden food rewards on the cheeseboard maze using distal cues ("allocentric learning condition") as described previously (Dupret et al., 2010). Briefly, each daily experiment consisted of a sequence of five recording sessions in the following order: a probe test ("pre-probe"), an immobility/sleep rest session ("pre-sleep"), a learning session, an immobility/sleep rest session ("post-sleep") and a probe test ("post-probe") (see **Figure S1**). Hippocampal neuronal assembly activity was continuously monitored during these sessions. The two probe sessions (~25 min) were never rewarded. After both the pre-probe and the learning sessions, rats were allowed to settle down within the start box for the rest sessions (~25 min). During the learning session, rats were given successive trials (~40 trials) to locate a new set of 3 hidden rewards placed in randomly selected food-wells every day. As these baited locations changed from day-to-day but stayed fixed within a given day, this "matching-to-multiple-places" procedure required frequent updating of memory for goal locations in an otherwise unchanging environment. To prevent the use of an odor-guided search strategy during these experiments, food pellet dust was scattered across the maze before each experiment, the board was periodically wiped (using the towel used to handle the rat daily) and the board was rotated relative to the start-box between learning trials and between rest and probe sessions. Rats were also trained to retrieve food rewards from visually marked goal locations ("cued learning condition")(Dupret et al., 2010). To do so, three identical objects (Falcon tube 50 ml, Greiner, see **Figure S2A**) were placed near the baited food-wells during the learning trials and rats were trained to retrieve the hidden rewards from these locations. In this condition, the task was solved by using a guidance strategy that consisted of moving towards intra-maze cues identified to be closely associated with the goals. In all, a total of 25 (from 9 animals) and 9 (from 4 animals) recordings sessions were analyzed for the "allocentric learning" and the "cued learning" conditions respectively, all incorporating sequences of probe-sleep-learning-sleep-probe sessions recorded in the familiar recording room. In three animals were recorded all two experimental conditions. In one animal, recordings were performed in the cued condition only because the quality of unit recordings deteriorated before recordings could be performed in the other condition. Learning performance and

locomotor speed were assessed off-line by calculating the distance travelled and the time spent to retrieve all three rewards during each trial. After completion of the experiments, the rats were deeply anesthetized and perfused through the heart with 0.9% saline solution followed by a 4% buffered formalin phosphate solution for the histological verification of the electrode tracks.

3. Supplemental References

Dupret,D., O'Neill,J., Pleydell-Bouverie,B., and Csicsvari,J. (2010). The reorganization and reactivation of hippocampal maps predict spatial memory performance. Nat.Neurosci. *13*, 995-1002.

Fisher,R.A. (1921). On the probable error of a coefficient of correlation deduced from a small sample. Metron *1*, 3-32.

Gilbert,P.E., Kesner,R.P., and DeCoteau,W.E. (1998). Memory for spatial location: role of the hippocampus in mediating spatial pattern separation. Journal of Neuroscience *18*, 804-810.

Kesner,R.P., Farnsworth,G., and Kametani,H. (1991). Role of parietal cortex and hippocampus in representing spatial information. Cereb.Cortex *1*, 367-373.

O'Neill,J., Senior,T., and Csicsvari,J. (2006). Place-selective firing of CA1 pyramidal cells during sharp wave/ripple network patterns in exploratory behavior. Neuron *49*, 143-155.

O'Neill,J., Senior,T.J., Allen,K., Huxter,J.R., and Csicsvari,J. (2008). Reactivation of experience-dependent cell assembly patterns in the hippocampus. Nat.Neurosci. *11*, 209-215.

Zar,J.H. (1999). Biostatistical Analysis. (Upper Saddle River: Pearson Education).
